# Manifold Learning Uncovers Nonlinear Interactions between the Adolescent Brain and the Social Environment in Predicting Mental Health Problems

**DOI:** 10.1101/2024.02.29.582854

**Published:** 2024-03-06

**Authors:** Erica L. Busch, May I. Conley, Arielle Baskin-Sommers

**Affiliations:** 1. Yale University, Department of Psychology, New Haven, CT, USA

**Keywords:** manifold learning, social environment, brain function, mental health, adolescent

## Abstract

Advanced statistical methods that capture the complex interplay between adolescents and their social environments are essential for improving our understanding of how differences in brain function contribute to mental health problems. To move the study of adolescent mental health beyond what we have achieved so far–a complex account of brain and environmental risk factors without understanding the neurobiological embedding of the social environment–we need to find ways of studying the complex, nonlinear relationships between brain function and adolescents’ experiences in the real-world. Manifold learning techniques can discover and highlight latent structure from high-dimensional, complex biomedical data, such as fMRI. Here, we develop a novel manifold learning technique, *exogenous* PHATE (EPHATE), to capture the interplay between brain function and adolescents’ social environments. By applying EPHATE, we demonstrate that harmonizing cutting-edge computational methods with longstanding developmental theory can advance efforts to detect and predict mental health problems during the transition to adolescence.

Nearly 75% of all mental health disorders onset during adolescence, with half of all mental health disorders occuring by age 14^1^. Adolescents who experience mental health problems are at heightened risk for lifelong challenges including lower educational attainment, increased legal system involvement, and chronic physical and mental health problems^2^. Developmental scientists have long grappled with trying to understand how mental health problems emerge in adolescents.

Thoughtful theories^3,4^ and empirical research provide a foundation for understanding factors related to mental health problems in adolescents. Much of this work has been separated into research specifying the neurobiology of mental health problems or the role of the social environment in the development of mental health problems. For example, convergent findings suggest that three key brain regions are especially sensitive during adolescent development: prefrontal cortex (PFC), amygdala (AMY), and hippocampus (HIP)^5^. These brain regions support self-regulation and affective processing^6,7^, and differences in the functional activation of these regions has been related to mental health problems^5^. However, recent studies question the reliability of associations between brain function and mental health problems in adolescents^8,9^, especially in the absence of accounting for adolescents’ experiences in the social environment^10,11^, which, by themselves, can be risk factors for mental health problems^12^. Specifically, meta-analyses show medium to large effects between adversity in adolescents’ families (e.g., conflict, caregiver nonacceptance) and neighborhoods (e.g., experiencing violence or disadvantage) and mental health problems^13–15^. Yet, there is substantial variability in how adolescents respond to their environments. Thus, increasingly, researchers are examining the complex interactions between adolescent neurobiology and the social environment.

The interaction between adversity in adolescents’ social environment and brain function is supported in the broadest sense with a few studies testing these multiple components simultaneously. For instance, a recent study found that the interaction of neighborhood adversity and lower executive network activation during an emotional working memory task was related to higher externalizing problems in adolescents^16^. Another study found that different forms of environmental adversity, in the family and neighborhood, interacted with functional connectivity in brain networks spanning PFC to predict internalizing symptoms in adolescents^17^. There also is evidence that the interaction between neighborhood adversity and decreased AMY activation during an emotional introspection task was related to higher externalizing problems in a sample of Mexican-origin adolescents^18^. Across these studies, we can stitch together a compelling conceptual model of mental health problems that includes interactions between experiences in adolescent’s social environments and brain function in regions involved in emotional and behavioral regulation. However, the majority of previous research investigated adolescent’s social environments and brain function as a linear interaction between univariate measures of brain and environment, which fails to account for the nonlinear, multidimensional interplay between adolescents’ developing brains and the social environment in which these brains are embedded.

To move closer to the goal of examining the nonlinear, multidimensional interplay between adolescent’s social environments and developing brain as an influence on mental health problems, statistical methods that can combine and identify structure in high-dimensional, multivariate data are needed. Manifold learning, a nonlinear approach to dimensionality reduction, is increasingly popular for highlighting complex latent structure in high-dimensional biological data^19^. In particular, PHATE, an unsupervised manifold learning algorithm designed for high-dimensional, noisy biomedical data, has been used to discover local and global latent structure in functional magnetic resonance imaging (fMRI) data^20–22^. Prior research showed that combining PHATE with additional data (e.g., stimulus structure or temporal dynamics of brain responses) can enhance the relevance of the latent structure of brain activation for understanding complex cognitive processing, for example during movie-viewing^23^. However, it remains unclear whether PHATE can 1) be used to enhance the behavioral relevance of task-based, developmental fMRI data during cognitive and emotional processing and 2) combined with additional information about social environments to discover latent geometric structure among adolescents’ social environments, brain function, and mental health problems.

Here, we examined whether integrating manifold learning and measures of family and neighborhood factors could enhance our ability to relate brain function to mental health problems during adolescence. We leveraged the Adolescent Brain Cognitive Development^SM^ Study (ABCD Study^®^) sample and longitudinal data. First, we applied the PHATE manifold learning algorithm to model patterns of brain activation during cognitive and emotion processing in an emotional *n*-back task (EN-back)^24^ collected at the baseline visit when youth were ages 9–10, and tested whether PHATE enhanced our ability to detect brain activation related to task performance. We hypothesized that the latent structure discovered within the PHATE manifold would have stronger associations with EN-back task performance relative to the voxel data. Next, we combined the PHATE brain activation manifold with measurements of adolescents’ social environments (also from baseline) into a *multi-view* manifold to evaluate whether the interplay between social environments and brain functioning enhanced our ability to detect mental health problems during the transition to adolescence. Multi-view approaches combine different measurements or data types collected from the same samples into a single representation to be embedded in lower dimensions. For instance, temporal PHATE (T-PHATE) is a recently-introduced multi-view manifold learning algorithm which combines two signals *endogenous* to brain data (i.e., calculated directly from the fMRI measurements). In the present study, we introduce *exogenous* PHATE (EPHATE), which combines participants’ PHATE brain activation manifold with data about the same participants collected *external* to their brain responses (Figure 1A). We hypothesized that using EPHATE to combine brain activation data with information about adolescents’ families and neighborhoods would show a stronger relationship with mental health problems both cross-sectionally and longitudinally^3,25–27^ (i.e., two years later, when youth were ages 11–12) than either the PHATE or voxel data. Overall, we show that EPHATE embeddings capture nonlinear interactions between brain function and the social environments, which, in turn, improved the detection and prediction of mental health problems in adolescents.

## Results and Discussion

### PHATE strengthened associations between brain activation and task performance

**Figure 1:**
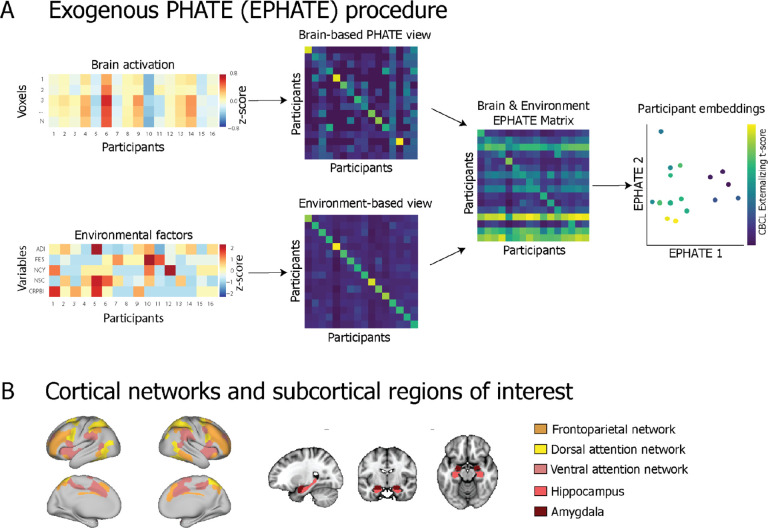
Exogenous PHATE (EPHATE) procedure **A:** EPHATE models the interactions between brain activation and exogenous information about participants using multi-view manifold learning. Here, the first view of EPHATE takes as inputs a vector of voxel-wise beta values from a given region of interest for each participant in the sample and learns a PHATE-based affinity matrix between each participants’ brain activations.The second view takes a vector of social environment scores for each of those participants and builds an affinity matrix across those scores, which is then row-normalized to become a transition probability matrix. These two views are then combined into one matrix (the EPHATE diffusion operator) which jointly characterizes both the brain and environment manifold geometry. This matrix is then embedded into lower dimensions using metric multidimensional scaling (m-MDS). Participants’ coordinates in EPHATE dimensions visually reflect individual differences along mental health metrics (here, externalizing problem scores). Main analyses presented here include 5 factors of social environment: ADI = area deprivation index, a composite index of neighborhood disadvantage; FES = family conflict; NCY = youth perceived neighborhood safety/crime; NSC = caregiver perceived neighborhood safety/crime; CRPBI = caregiver acceptance **B:** Subsequent analyses are presented using beta values extracted for voxels in the bilateral amygdala and hippocampus and surface vertices in three cortical networks: frontoparietal, dorsal and ventral attention networks.

We first validated that PHATE embeddings of regional brain activity correlated with EN-back task performance. The EN-back task measures memory and emotion processing via the linear contrasts of 2-back vs. 0-back blocks and emotion vs. neutral face stimulus blocks, respectively^24^. For each participant (*n* = 5,235), we extracted beta weights of the 2-back vs. 0-back and emotion vs. neutral face contrasts from all voxels in five regions of interest (ROIs): AMY, HIP, frontoparietal network, dorsal and ventral attention networks (Figure 1B). This produced one participants-by-voxels matrix for each region and contrast. We then embedded each activation matrix using PHATE to get a comparable participants-by-PHATE components matrix. Using a cross-validated linear regression, we measured associations between task performance and brain activation using voxel resolution patterns and corresponding PHATE embeddings. Relationships between brain activation and EN-back performance were assessed by correlating the model’s prediction of task performance on held-out data with true performance. Models trained on voxel data and PHATE embeddings were compared using a two-tailed permutation test (10,000 iterations) with Bonferroni correction.

**Figure 2:**
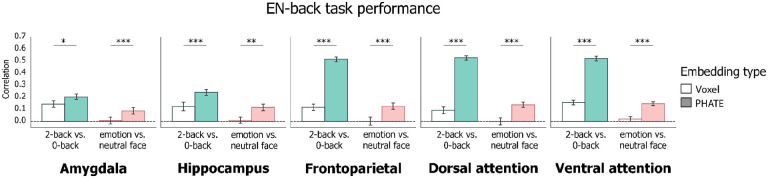
Associations of brain data with EN-back task performance Bars represent average correlation between model predicted and true EN-back scores on held-out participants’ data. Left column in each graph represents the 2-back vs. 0-back contrast; Right column in each graph represents the emotion vs. neutral contrast. Error bars represent the 95% confidence interval of the mean across 20 cross-validation folds. ~ p < 0.1, * p < 0.05, ** p < 0.01, *** p < 0.001

In all regions evaluated, regression models trained on the PHATE embeddings significantly outperformed those trained on the voxel data (Figure 2, Table 2; Extended Data 2). PHATE embeddings from the 2-back vs. 0-back contrast were significantly related to EN-back task performance in all regions, with the largest magnitude of effects (*r* > .50) in the frontoparietal and attention networks. In comparison, voxel data in response to the 2-back vs. 0-back contrast showed smaller (*r* < 0.20) associations with EN-back performance in all regions (Table 2). Given previous research linking frontoparietal and attention networks with higher order cognitive abilities and working memory^28–30^, these results demonstrate that PHATE optimized the utility of fMRI data for detecting behaviorally relevant brain activation during cognitive processing.

Further, PHATE embeddings from the emotion vs. neutral contrast showed moderate associations with EN-back performance (*r* > 0.12 in frontoparietal and attention networks). In contrast, and consistent with prior research^28^, none of the voxel data for the emotion vs. neutral face contrast significantly related to EN-back performance. Therefore, embedding brain activation with PHATE enhanced the relevance of emotion processing-related brain activation for understanding performance during the EN-back. Together, these results demonstrate that the manifold learning algorithm PHATE may be useful for optimizing fMRI data to understand how brain function relates to cognitive and emotional processing.

### EPHATE enhanced cross-sectional associations with mental health problems

We designed EPHATE to model the nonlinear interaction between brain activation and exogenous information about the social environments. The first view of EPHATE modeled PHATE-based affinities between participants’ brain activity patterns, and the second view modeled affinities between participants’ social environments. These two views jointly characterized brain- and social-environmental manifold geometry, which was then embedded into lower (here, 20) dimensions. We then tested whether EPHATE strengthened associations between brain function and mental health problems (using scores from the Child Behavior Checklist [CBCL]^31^).

**Figure 3:**
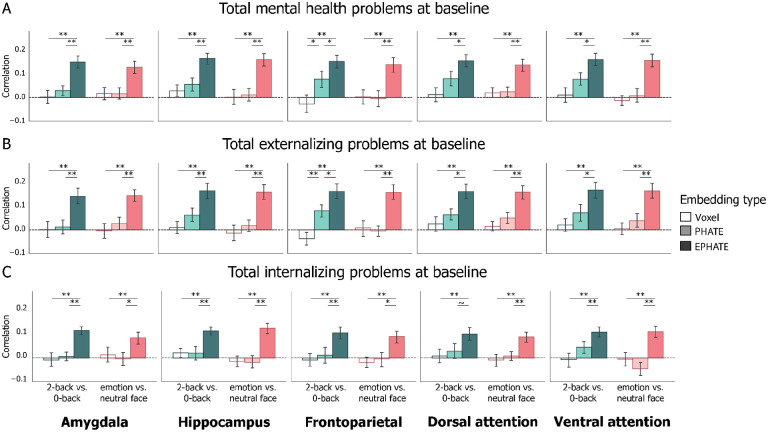
Cross-sectional associations of brain data with mental health problems Bars represent average correlation between model predicted and true mental health problem scores on held-out participants’ data, trained and tested with 20-fold cross validation. Both mental health scores and brain/environmental data were collected at the baseline timepoint. Left column in each graph represents the 2-back vs. 0-back contrast; Right column in each graph represents the emotion vs. neutral contrast. A=Total problems; B=Externalizing problems; C=Internalizing problems. Error bars represent the 95% confidence interval of the mean across 20 cross-validation folds. ~ p < 0.1, * p < 0.05, ** p < 0.01, *** p < 0.001

Voxel activity from the 2-back vs. 0-back working memory contrast significantly related to total mental health problems above chance in only the hippocampus (Table 2). PHATE embeddings of the 2-back vs. 0-back contrast were significantly more related to total mental health problems than the voxel data in only the control network. As expected, EPHATE reflected stronger associations between brain activation and total mental health problems relative to voxel data and PHATE embeddings for the 2-back vs. 0-back contrast for every region (all p’s < 0.05; Figure 3A; Extended Data 2).

Replicating previous research showing null associations between emotion-processing activation and mental health problems^32^, voxel activity from the emotion vs. neutral contrast did not significantly relate to individual differences in total mental health problems in any ROI. Without added information about the social environment, PHATE embeddings of the emotion vs. neutral contrast performed similarly to the voxel data in the magnitude of effect with total mental health problems. EPHATE significantly enhanced the relationship between brain activation and total mental health problems relative to the voxel data and PHATE embeddings in the emotion vs. neutral contrasts for all regions (all p’s < 0.01; Figure 3A; Table 2; Extended Data 2).

The CBCL total problem score can be further broken into two main broad-band scales: externalizing and internalizing problems. To examine whether the significant relationships between EPHATE embeddings and total mental health problems were driven by associations with externalizing or internalizing problems, we repeated the analyses above testing the correlations with each of the broad-band CBCL scales separately. As above, results demonstrated that EPHATE embeddings outperformed the voxel data or PHATE embeddings across both task contrasts in all ROIs (all p’s < 0.05; Figure 3B; Extended Data 2). The magnitude of effects for internalizing problems was generally lower than that of externalizing problems (Figure 3C; Table 2). Supplementary analyses revealed that this pattern replicated across the externalizing and internalizing subscales as well (Supplementary Figure 1; Extended Data 1).

To confirm that the increased sensitivity of EPHATE for detecting mental health problems was attributable to added information specifically about the social environment, as opposed to an increase in the sheer quantity of information provided about each participant, we replaced the social environment variable matrix with other exogenous information about participants that should be irrelevant to their mental health, including height, weight, handedness, number of siblings, and age (in months). Results from this sensitivity analysis demonstrated that adding the same quantity of information into EPHATE did not enhance associations with mental health problems (Supplementary Figure 2; Extended Data 2).

In addition, we asked whether specific variables about the social environment were more valuable than others in enhancing the sensitivity of EPHATE to mental health problems. We considered second views of the EPHATE matrix built solely upon neighborhood disadvantage and family conflict , which are commonly considered in isolation as measures of environmental adversity^33,34^. While adding either neighborhood disadvantage or family conflict alone did improve performance, neither variable afforded as great an improvement as the five-feature social environment view, further emphasizing the importance of a multidimensional interplay between different aspects of the social environment and brain function^12^ (Supplementary Figure 3).

Altogether, EPHATE provided a richer representation of memory- and emotion-processing activation, which uncovered latent structure in patterns of brain activation relevant for detecting cross-sectional relationships with mental health problems. These results demonstrate that efforts to elucidate relationships between adolescent brain function and mental health problems may be stifled if researchers fail to consider the broader context in which brain development is embedded^27^. To optimize neuroimaging data to understand mental health problems, researchers may benefit from incorporating statistical methods, such as EPHATE, which are suited to model complex, nonlinear interactions between adolescents’ neurobiology and their social environments.

### EPHATE improved longitudinal prediction of mental health problems

Finally, to test whether EPHATE could enhance our ability to detect brain activation relevant for predicting future mental health problems, we asked whether the same latent space built from brain and social environmental factors at baseline (ages 9–10) could predict mental health problems two years later (at ages 11–12). Using a subset of the original participants (*n* = 2,686 with complete 2-year follow-up data), we embedded baseline brain activation data and social environment factors with EPHATE and used linear regression to predict total mental health, externalizing, and internalizing problems two years later.

**Figure 4:**
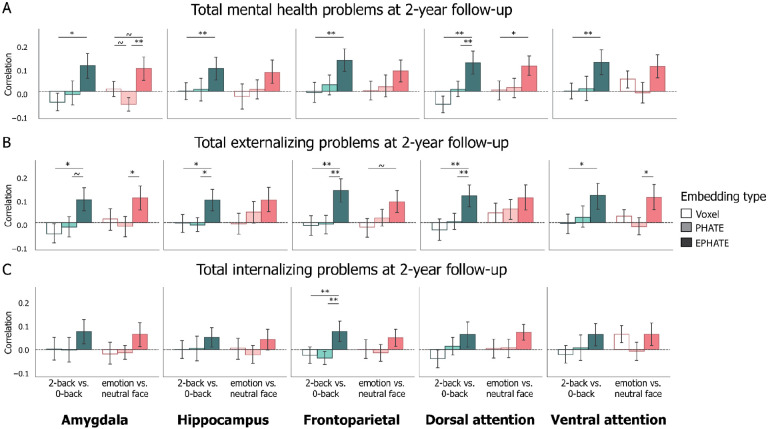
Longitudinal prediction of mental health problems Bars represent average correlation between model predicted and true mental health problem scores on held-out participants’ data, trained and tested with 20-fold cross validation. Brain and environment data were collected from the baseline timepoint, and mental health scores were collected at the 2-year follow-up. Left column in each graph represents the 2-back vs. 0-back contrast; Right column in each graph represents the emotion vs. neutral contrast. A=Total problems; B=Externalizing problems; C=Internalizing problems. Error bars represent the 95% confidence interval of the mean across 20 cross-validation folds. ~ p < 0.1, * p < 0.05, ** p < 0.01, *** p < 0.001

The effect sizes for longitudinal prediction were generally lower for all regions and contrasts relative to the cross-sectional results described above, however, EPHATE embeddings for several contrasts and regions significantly predicted mental health problems two years later (Table 2; Extended Data 1). Specifically, EPHATE embeddings from the 2-back vs. 0-back contrast better predicted total problem and externalizing scores from HIP and AMY (p’s < 0.05) and frontoparietal and attention network activation (p’s < 0.01) relative to voxel resolution data. EPHATE embeddings from the 2-back vs. 0-back contrast also predicted total problem and externalizing scores from dorsal attention network activation and predicted externalizing scores from frontoparietal network activation significantly better than PHATE embeddings (Figure 4A; Extended Data 2). As with the cross-sectional analysis, longitudinal prediction of internalizing scores was lower compared to total problem or externalizing scores (Figure 4B). Internalizing problems were only significantly predicted by EPHATE embeddings from the 2-back vs. 0-back contrast of frontoparietal network activation (Figure 4C; Table 2; Extended Data 1).

Consistent with previous research linking aberrant AMY function during emotion processing with mental health problems^35,36^, EPHATE embeddings from the emotion vs. neutral contrast predicted total and externalizing problems from AMY activation better than PHATE embeddings. EPHATE embeddings from the emotion vs. neutral contrast also predicted externalizing problems from dorsal attention activation (Figure 4B; Table 2). Overall, the latent structure of memory and emotion processing-related brain activation captured with EPHATE may increase the application of fMRI data for efforts to identify early markers of risk for future mental health problems in adolescents.

### Summary and future directions

We found that manifold learning enhanced the relevance of task-based fMRI data for understanding behavioral performance and mental health problems in a large, sociodemographic diverse sample of US adolescents. Specifically, we demonstrated that applying PHATE to uncover the low-dimensional structure in brain activation data during cognitive and emotion processing improved associations with task performance. Further, we introduced EPHATE, a multi-view manifold learning approach to integrate brain activation data with exogenous measures. Modeling nonlinear interactions between brain function and the social environment allowed for greater detection and prediction of mental health problems during adolescence. Overall, our results demonstrate that manifold learning techniques are well-suited for the complexity of developmental fMRI data and have great potential to enhance research on the neurobiology of mental health problems in adolescents.

A major goal of developmental science is to represent the complex interplay between youth and their broader social environments^3,16,18,25–27^. Here, we make a substantial methodological advance through the development of EPHATE. First, EPHATE can model the nonlinear interplay between adolescents’ neurobiology and experiences throughout their social environments, offering future researchers an alternative to linear approaches that model interactions as a simple product of two variables^37^. Second, by incorporating exogenous information about adolescents’ neighborhoods and families as essential data adding structure to the manifold, EPHATE improved associations between brain activation and mental health problems. Previous studies have questioned the reliability of empirical support linking specific ROIS (e.g., AMY) to mental health problems in youth^8,9^, yet EPHATE highlighted signals relevant for understanding individual differences in mental health problems in every ROI and network we examined across both contrasts. Thus, the current study demonstrates that multi-view manifold learning techniques such as EPHATE may be especially useful for understanding how the complex interplay between adolescent neurobiology and the social environment in which the brain is embedded relate to mental health problems in adolescents.

The present work should be viewed in light of a few limitations. First, we focused on specific ROIs and networks that have previously been related to memory- and emotion-processing and mental health^5,6,24,28–30^. Yet, investigating other brain areas or whole-brain approaches may be relevant for understanding task performance and mental health problems. Second, manifold learning algorithms are not able to discern the direction or specific patterns of brain activation that contribute to associations with task performance and mental health problems. Third, while EPHATE could predict mental health problems two years later, results from the current study are correlational and cannot speak to causation. In light of research showing bidirectional relationships between the social environment and mental health problems^38,39^, future studies should incorporate manifold learning within other longitudinal designs. Fourth, the results in the current paper only reflect a snapshot of development. Given that the peak onset of mental health problems is later in adolescence^1^, future research investigating a larger developmental window is needed. Nevertheless, results from the current study introduce two novel applications of manifold learning to optimize research on the neurobiology of adolescent mental health.

## Conclusion

EPHATE represents a major methodological innovation in our ability to harmonize computational advances for complex biomedical data with longstanding developmental theory to progress our understanding of mental health problems. The current study advances empirical support linking the interplay between neurobiology and the social environment with mental health problems in adolescents. Ultimately, data-driven, interdisciplinary approaches that characterize adolescents’ changing neurobiology within the context of their social environments may allow us to identify early markers of risk and novel targets for intervention.

## Methods

### Participants

Participants were adolescents included in ABCD Data Release 4.0 (DOI:10.15154/1523041). The ABCD Study is a longitudinal study following youth from 21 sites across the United States^40^. Study procedures received centralized University of California, San Diego and site-specific institutional review board approval^41^. Parents or caregivers provided written informed consent and adolescents gave written assent for study participation. ABCD study-wide exclusion criteria included a diagnosis of schizophrenia, moderate to severe autism spectrum disorder, intellectual disability, substance use disorder at recruitment, persistent major neurological disorders, multiple sclerosis, sickle cell disease, seizure disorders like Lennox-Gastaut Syndrome, Dravet Syndrome, and Landau Kleffner Syndrome. Here, we analyzed environmental, mental health, and fMRI data from the baseline assessment when adolescents were ages 9–10 and mental health data from the 2-year follow up when adolescents were ages 11–12. Additional participant exclusions were made based on missing fMRI, environmental, or mental health measures, resulting in 5,235 participants included in baseline analyses. Of those, 2,686 participants had complete data at the two-year follow up. Sample demographics are detailed in Table 1.

### Social environment measures

Measures of the social environment were selected to characterize youth’s family and neighborhood social environments using baseline assessments from the ABCD Study Culture and Environment^36^ and Linked External Data protocols.

Family threats were measured with the youth-report ABCD Family Environment Scale–Family Conflict Subscale, which included nine items measuring youth’s perception of anger and conflict expressed among family members. For each item, youth endorsed whether a statement was true or false for most family members. All items were summed with higher scores indicating *more* family conflict.

Family support and acceptance was assessed with the Children’s Report of Parental Behavioral Inventory, which included five items measuring youth’s perception of caregiver acceptance and support. For each item, youth indicated whether a statement was not, somewhat, or a lot like their primary caregiver. All items were averaged and reverse scored such that higher scores indicated *less* family support/acceptance.

Neighborhood threats were measured using the ABCD Neighborhood Safety/Crime Survey, which assessed perceived safety/crime in participants’ neighborhoods. Because the adolescent assessment included only a single item asking youth whether they strongly agreed or strongly disagreed with a statement about their safety from neighborhood crime, neighborhood threats were measured as the mean of all adolescent- and caregiver-report items. The caregiver survey included the exact item from the youth survey with two other items asking whether caregivers strongly agreed or strongly disagreed with statements about their perceived safety while walking and violence being a problem in their neighborhood. All adolescent- and caregiver-report items were reverse-scored such that higher scores indicated *more* neighborhood threats.

Neighborhood deprivation was measured using the Area Deprivation Index (ADI), which is a composite index of neighborhood socioeconomic disadvantage based on income, education, employment and housing quality in a census tract derived from the American Community Survey. Participants ADI national percentile were calculated for their primary address, and higher scores indicated *more* neighborhood deprivation.

### Mental health measures

Measures of adolescent mental health problems were assessed using *t*-scores from the baseline and two-year follow-up data from the Achenbach System of Empirically Based Assessment Child Behavior Checklist (CBCL), which is a 119 item parent/caregiver-report survey of adolescent mental health problems validated for use in adolescents ages 6–18^42^. Primary analyses examined total problems, and externalizing, and internalizing broad-band scales, and supplemental analyses examined anxious/depression, withdrawn/depression, aggression, and rule-breaking behavior syndrome scales.

### Emotional n-back task

The in-scanner emotional n-back (EN-back) task was designed to engage emotion and memory processing^40,43^. The task included two ~5 minute fMRI runs, each with eight task blocks and four 15s fixation blocks. During each run, participants performed four 0-back (low memory load) and four 2-back (high memory load) blocks with happy, fear, or neutral face or place stimuli. For each trial, stimuli were presented for 2s and followed by 500ms fixation cross. On 0-back blocks, participants were instructed to press “match” when the stimulus was the same as a target stimulus presented at the beginning of the block, and “no match” if not. On 2-back blocks, participants were instructed to press “match” when the stimulus was the same as the stimulus presented two trials back, and “no match” if not. EN-back behavioral performance was measured with sensitivity, calculated as d’ = z(hits) - z(false alarms) and adjusted for extreme values^44^.

### Neuroimaging data

fMRI data were collected on Siemens Prisma, Phillips, and GE 750 3T scanners using a 32-channel head coil^24^. Functional images were collected with a multiband gradient echo-planar imaging sequence and the following parameters: TR = 800 ms, TE = 30 ms, flip angle = 52°, 60 slices acquired in the axial plane, voxel size = 2.4mm^3^, multiband slice acceleration factor = 6. All fMRI data were preprocessed by the ABCD Study Data Analysis, Informatics, and Resource Center (DAIRC) as detailed elsewhere^45^.

Following preprocessing, EN-back activation was estimated for each participant using general linear models with fixation, 2-back and 0-back condition, and happy, fearful, and neutral face and place stimuli as predictors^45^. We evaluated emotion processing activation with the linear contrast of emotional and neutral face task blocks and cognitive processing activation with the linear contrast of 2-back and 0-back task blocks. Participants were excluded based on low quality structural scans, fewer than 550 degrees of freedom in preprocessed, concatenated timeseries data, task contrast maps missing over 260 grayordinate values (e.g., missing values outside the medial wall), and extreme values (> 3 SD from the group mean) for the mean or SD of beta weights across all grayordinates, as in prior studies^16^.

For the two EN-back contrasts (2-back vs. 0-back and emotion face vs. neutral), we analyzed beta weights from networks defined using the Schaefer parcellation^46^, which delineates 400 cortical parcels within 7 networks^47^, and the Scale I Tian subcortical parcellation^48^, which delineates 8 bilateral subcortical regions. Multivoxel activation patterns were extracted from three networks (frontoparietal, dorsal attention, and ventral attention networks) and two bi-lateral subcortical structures (amygdala and hippocampus). Activation patterns were then vectorized, resulting in one vector for each region, participant, and contrast.

### Manifold embeddings

Manifold learning was used to test whether a low-dimensional embedding of participants’ brain activation patterns better captured behaviorally-relevant activation during cognitive and emotion processing. In particular, we applied the PHATE algorithm, a diffusion-based manifold learning method developed for performing dimensionality reduction on high-throughput biomedical data. PHATE captures local neighborhoods in the data using a diffused Markov transition matrix and also maintains global data context using a potential distance metric. This allows PHATE manifolds to reflect local and global manifold structure and perform effective denoising and makes PHATE well suited for the high dimensionality and intrinsic noise of fMRI activity^21,23^.

Given PHATE’s suitability for learning faithful data manifolds from fMRI data, we expected individual differences in fMRI activation could be summarized in a low-dimensional subspace, and this subspace would in turn better relate to EN-back task performance and mental health problems compared with voxel data. To examine this, we first extracted beta weights from five regions of interest during the emotion vs. neutral face and 2-back vs. 0-back contrasts. This resulted in a participants-by-voxels matrix for each region and contrast, which was then embedded using PHATE into 20 dimensions to get a participants-by-20 PHATE dimensions matrix. Dimensionality was selected based on prior literature^21,23^ and was kept consistent across regions, contrasts, and embedding methods.

Next, we examined whether combining PHATE with additional information about adolescent’s social environments better captured brain activation patterns relevant for uncovering associations with mental health problems. In previous work, we found that fMRI timeseries data was best captured using a manifold that combined PHATE manifold geometry and fMRI temporal dynamics. Temporal PHATE (T-PHATE) uses a dual-diffusion process to combine two endogenous, interacting signals in neuroimaging data: activation across space and activation which unfurls over time^23^. In the current study, we applied a similar dual-diffusion approach to combine exogenous information about adolescents’ social environments with brain activation. Thus, we designed the *exogenous* PHATE (EPHATE) procedure, which combines the PHATE diffusion matrix and a second view based on an affinity matrix over participants’ scores on additional, external variables (here, environmental factors).

Given a matrix *X* =*x*_1_, *x*_2_, *x*_*n*_, where $x_{n}\in {\mathbb{R}}^{N}$ for participant *n* is a *V*-dimensional vector, and *V* is the number of voxels or vertices in a ROI. The construction of the PHATE diffusion geometry is summarized as steps 1–4 here and described in more detail in^20,23^:

1. Calculate euclidean distance matrix between data pairs (pairs of participants), where:
$$D(i,j)={\left\|x_{i}-x_{j}\right\|}^{2}$$
2. Convert D from a distance matrix into a local affinity matrix K using an adaptive bandwidth Gaussian kernel.
$$𝒦(x,y)=\frac{𝒢(x,y)}{∥𝒢(x,\cdot ){∥}_{1}^{\alpha }∥𝒢(y,\cdot ){∥}_{1}^{\alpha }},\;𝒢(x,y)={\text{e}}^{-\frac{∥x-y{∥}^{\alpha }}{\sigma }}$$
3. Row-normalize K to define transition probabilities into the *N* × *N* row stochastic matrix, *P*.
$$P(i,j)=\frac{𝒦\left(x_{i},x_{j}\right)}{∥𝒦(x,\cdot ){∥}_{1}}$$
4. Use probabilities P for a Markovian random-walk diffusion process and compute the diffusion timescale tD, which specifies the number of steps taken in the random walk, based on the von neumann entropy of the diffusion operator. Perform the tD-step random walk over P by raising P to the tD power. Then, compute the diffusion potential distance PD between all pairs of distributions at row i and row j:
$$P_{D}(i,j)=\sqrt{\sum_{k}(\text{log}{\left(P^{t_{D}}(i,k)-P^{t_{D}}(j,k)\right)}^{2}}$$
  Given a matrix *F* = *f*_1_, *f*_2_, … *f*_*n*_ where $f_{n}\in {\mathbb{R}}^{N}$ for participant *n* is a *k*-dimension vector, and *k* is the number of exogenous (matched samples of a different measurement) features one opts to include in the second view. The EPHATE procedure extends beyond the original PHATE formulation in two more steps:
5. Calculate the an affinity matrix *A* as the euclidean distance between data pairs (pairs of participants) across feature vectors, and normalize the value to be between 0 and 1, where 1 corresponds to maximal similarity:
$$A(i,j)=\frac{1}{1+\left\|f_{i}-f_{j}\right\|}$$
6. Convert the affinity matrix *A* into the transition probability matrix *P*_*A*_ by row-normalizing the affinity matrix A, as in step 3.
7. Combine *P*_*A*_ with the result of step 4 *P*_*D*_ via alternating diffusion:
$$P=P_{A}P_{T}$$
8. Embed with metric multidimensional scaling (MDS) into *M* dimensions, where *M* = 2–3 for visualization or higher for downstream analysis.

This dual-diffusion step allows EPHATE to learn data geometry across the neuroimaging dimensions (voxels or surface vertices) and across the dimensions of environmental metrics. Then, the dual-diffusion matrix is embedded with MDS scaling into 20 dimensions. For consistency, we compare EPHATE embeddings with 20-D PHATE embeddings and the full voxel resolution data.

### Associations with behavioral and mental health outcomes

To predict behavioral or mental health outcomes from neuroimaging data (either voxel resolution data or embeddings) we used a linear regression with 20-fold cross-validation. Cross-sectional association used participants’ brain data at baseline to predict their mental health scores at the baseline time point. Longitudinal prediction used participants’ brain data at baseline to predict their mental health scores at the 2-year follow-up. Longitudinal prediction only trained on participants who had both baseline and 2-year follow up data (*n*=2,686) whereas the cross-sectional association included participants with complete baseline data (*n*=5,235). Regression models were scored as the Pearson correlation coefficient between model predicted values and true values on held-out data. Results are presented with bars as the average prediction accuracy across cross-validation folds and error bars representing the 95% confidence interval calculated with 1,000 bootstrap samples.

Statistical significance of the difference in prediction performance between pairs of methods was assessed using a permutation test as follows. For each pair of methods, within ROI and contrast, we calculated the difference in cross-validation scores across the two methods, within folds to preserve participant-wise random effects. We then generated a null distribution of difference scores across the two methods by randomly permuting the method label of a fold’s score 10,000 times and recomputing the mean difference between the methods, then computing a two-tailed p-value of the true difference relative to the null distribution. P-values were then corrected for multiple comparisons using the Bonferroni method, within each contrast but across the 5 ROIs.

## Supplementary Material

1

## Figures and Tables

**Table 1.** Demographics for participants included in the baseline (ages 9–10) and 2-year follow-up (ages 11–12) analyses.

Timepoint	Baseline	2-year follow-up
**n (percent)**	5235	2686
**Female**	2672 (51)	1338 (50)
**Race/Ethnicity**		
Hispanic or Latinx	942 (18.0)	477 (17.8)
Black	535 (10.2)	240 (8.9)
White	3076 (58.8)	1654 (61.6)
Asian	113 (2.2)	52 (1.9)
Other	569 (10.9)	263 (9.8)
**Caregiver education (%)**		
< HS Diploma	160 (3)	77 (2.9)
HS Diploma / GED	366 (6.9)	156 (5.8)
Some College	1194 (22.8)	628 (23.4)
Bachelor	1422 (27.2)	730 (27.2)
Post Graduate Degree	2090 (39.9)	1091 (40.6)
Unknown	3 (0.05)	4 (0.1)
**Family income (%)**		
<$50K	1116 (21.3)	566 (21.1)
> $50K - < $100K	1435 (27.4)	1166 (43.4)
>$100K	2333 (44.6)	786 (29.3)
Unknown	351 (6.4)	168 (6.2)

**Table 2.** Results showing associations between brain activation embeddings and behavioral performance and mental health problems.

Embedding type	ROI	Pearson’s r (baseline)	95% CI (baseline)	Pearson’s r (2-year)	95% CI (2-yearr)
**EN-Back performance**					
2-back vs. 0-back					
voxel	Amygdala	0.145	[0.116, 0.172]	n/a	
	Hippocampus	0.121	[0.088, 0.164]		
	Dorsal attention network	0.093	[0.066, 0.177]		
	Ventral attention network	0.157	[0.088, 0.164]		
	Frontoparietal network	0.117	[0.090, 0.144]		
PHATE	Amygdala	0.203	[0.182, 0.228]		
	Hippocampus	0.241	[0.219, 0.265]		
	Dorsal attention network	0.528	[0.511, 0.546]		
	Ventral attention network	0.523	[0.504, 0.541]		
	Frontoparietal network	0.519	[0.498, 0.537]		
Emotion vs. neutral face					
voxel	Amygdala	0.007	[−0.032, 0.029]	n/a	
	Hippocampus	0.008	[−0.013, 0.031]		
	Dorsal attention network	0.001	[−0.030, 0.026]		
	Ventral attention network	0.019	[−0.002, 0.038]		
	Frontoparietal network	0.001	[−0.029, 0.036]		
PHATE	Amygdala	0.087	[0.063, 0.113]		
	Hippocampus	0.117	[0.089, 0.145]		
	Dorsal attention network	0.138	[0.118, 0.165]		
	Ventral attention network	0.147	[0.130, 0.164]		
	Frontoparietal network	0.127	[0.102, 0.156]		
**CBCL total mental health problems**					
		2-back vs. 0-back (baseline)		2-back vs. 0-back (2-year)	
voxel	Amygdala	0.002	[−0.030, 0.027]	−0.039	[−0.070, −0.006]
	Hippocampus	0.028	[0.004, 0.051]	0.001	[−0.028, 0.036]
	Dorsal attention network	0.012	[−0.016, 0.044]	−0.046	[−0.078, −0.018]
	Ventral attention network	0.011	[−0.021, 0.040]	0.001	[−0.026, 0.033]
	Frontoparietal network	−0.027	[−0.062, 0.011]	−0.003	[−0.037, 0.038]
PHATE	Amygdala	0.029	[0.007, 0.049]	−0.010	[−0.047, 0.040]
	Hippocampus	0.055	[0.030, 0.083]	0.008	[−0.031, 0.051]
	Dorsal attention network	0.081	[0.047, 0.107]	0.008	[0.047, 0.107]
	Ventral attention network	0.078	[0.052, 0.104]	0.011	[−0.018, 0.043]
	Frontoparietal network	0.078	[0.046, 0.108]	0.024	[−0.021, 0.057]
EPHATE	Amygdala	0.150	[0.125, 0.175]	0.095	[0.054, 0.146]
	Hippocampus	0.165	[0.141, 0.187]	0.085	[0.044, 0.123]
	Dorsal attention network	0.155	[0.131, 0.182]	0.106	[0.066, 0.158]
	Ventral attention network	0.160	[0.135, 0.188]	0.107	[0.061, 0.151]
	Frontoparietal network	0.153	[0.126, 0.179]	0.115	[0.077, 0.163]
		Emotion vs. neutral face (baseline)		Emotion vs. neutral face (2-year)	
voxel	Amygdala	0.018	[−0.007, 0.043]	0.009	[−0.013, 0.038]
	Hippocampus	0.001	[−0.029, 0.034]	−0.017	[−0.069, 0.025]
	Dorsal attention network	0.020	[0.000, 0.042]	0.005	[−0.030, 0.034]
	Ventral attention network	−0.013	[−0.033, 0.006]	0.046	[0.014, 0.075]
	Frontoparietal network	0.003	[−0.026, 0.032]	0.003	[−0.029, 0.038]
PHATE	Amygdala	0.017	[−0.006, 0.043]	−0.047	[−0.074, −0.026]
	Hippocampus	0.012	[−0.018, 0.039]	0.007	[−0.027, 0.040]
	Dorsal attention network	0.024	[0.003, 0.043]	0.015	[−0.026, 0.046]
	Ventral attention network	0.009	[−0.020, 0.041]	−0.005	[−0.045, 0.029
	Frontoparietal network	−0.005	[−0.036, 0.027]	0.018	[−0.017, 0.065]
EPHATE	Amygdala	0.129	[0.104, 0.154]	0.085	[0.044, 0.129]
	Hippocampus	0.160	[0.135, 0.186]	0.071	[0.035, 0.121]
	Dorsal attention network	0.137	[0.113, 0.162]	0.094	[0.062, 0.134]
	Ventral attention network	0.157	[0.130, 0.184]	0.091	[0.056, 0.146]
	Frontoparietal network	0.139	[0.110, 0.169]	0.076	[0.031, 0.115]
**CBCL externalizing problems**					
		2-back vs. 0-back (baseline)		2-back vs. 0-back (2-year)	
voxel	Amygdala	0.001	[−0.040, 0.032]	−0.042	[−0.073, −0.002]
	Hippocampus	0.009	[−0.017, 0.032]	−0.001	[−0.037, 0.034]
	Dorsal attention network	0.024	[−0.007, 0.053	−0.027	[−0.062, 0.019]
	Ventral attention network	0.020	[−0.009, 0.043]	−0.003	[−0.036, 0.034]
	Frontoparietal network	−0.037	[−0.059, −0.011]	−0.010	[−0.050, 0.028]
PHATE	Amygdala	0.012	[−0.014, 0.041]	−0.017	[−0.051, 0.020]
	Hippocampus	0.062	[0.034, 0.089]	−0.008	[−0.032, 0.022]
	Dorsal attention network	0.064	[0.039, 0.089]	0.004	[−0.024, 0.037
	Ventral attention network	0.071	[0.031, 0.104]	0.020	[−0.017, 0.061]
	Frontoparietal network	0.080	[0.053, 0.106]	−0.005	[−0.046, 0.025]
EPHATE	Amygdala	0.140	[0.108, 0.175]	0.084	[0.045, 0.130]
	Hippocampus	0.165	[0.131, 0.196]	0.084	[0.048, 0.124]
	Dorsal attention network	0.162	[0.133, 0.192]	0.099	[0.058, 0.144]
	Ventral attention network	0.167	[0.135, 0.201]	0.100	[0.047, 0.138]
	Frontoparietal network	0.162	[0.133, 0.195]	0.118	[0.075, 0.159]
		Emotion vs. neutral face (baseline)		Emotion vs. neutral face (2-year)	
voxel	Amygdala	−0.003	[−0.036, 0.024]	0.013	[−0.024, 0.051]
	Hippocampus	−0.015	[−0.046, 0.018]	−0.004	[−0.042, 0.037]
	Dorsal attention network	0.014	[−0.005, 0.033]	0.036	[0.000, 0.071]
	Ventral attention network	0.005	[−0.023, 0.027]	0.024	[0.000, 0.047]
	Frontoparietal network	0.007	[−0.028, 0.038]	−0.017	[−0.052, 0.013]
PHATE	Amygdala	0.025	[0.004, 0.056]	−0.013	[−0.050, 0.023]
	Hippocampus	0.016	[−0.010, 0.042]	0.038	[0.003, 0.079]
	Dorsal attention network	0.049	[0.026, 0.073]	0.051	[0.012, 0.085]
	Ventral attention network	0.038	[0.010, 0.070]	−0.016	[−0.045, 0.021]
	Frontoparietal network	−0.006	[−0.028, 0.016]	0.017	[−0.013, 0.052]
EPHATE	Amygdala	0.143	[0.121, 0.169]	0.091	[0.049, 0.137]
	Hippocampus	0.158	[0.129, 0.191]	0.084	[0.049, 0.141]
	Dorsal attention network	0.157	[0.132, 0.187]	0.092	[0.045, 0.138]
	Ventral attention network	0.165	[0.129, 0.194]	0.093	[0.048, 0.137]
	Frontoparietal network	0.157	[0.130, 0.191]	0.007	[0.037, 0.118]
**CBCL internalizing problems**					
		2-back vs. 0-back (baseline)		2-back vs. 0-back (2-year)	
voxel	Amygdala	−0.009	[−0.040, 0.019]	0.002	[−0.035, 0.041]
	Hippocampus	0.021	[0.003, 0.040]	0.001	[−0.032, 0.032]
	Dorsal attention network	0.008	[−0.023, 0.037]	−0.033	[−0.064, −0.001]
	Ventral attention network	−0.009	[−0.045, 0.020]	−0.018	[−0.051, 0.013]
	Frontoparietal network	−0.010	[−0.036, 0.017]	−0.021	[−0.048, 0.015]
PHATE	Amygdala	0.005	[−0.012, 0.023]	−0.001	[−0.041, 0.045]
	Hippocampus	0.019	[−0.009, 0.053]	0.004	[−0.035, 0.059]
	Dorsal attention network	0.027	[−0.004, 0.056]	0.013	[−0.020, 0.050]
	Ventral attention network	0.044	[0.018, 0.068]	0.006	[−0.042, 0.047]
	Frontoparietal network	0.010	[−0.020, 0.044]	−0.031	[−0.059, −0.009]
EPHATE	Amygdala	0.115	[0.100, 0.132]	0.065	[0.026, 0.117]
	Hippocampus	0.113	[0.095, 0.131]	0.045	[0.006, 0.081]
	Dorsal attention network	0.101	[0.077, 0.130]	0.056	[0.010, 0.100]
	Ventral attention network	0.109	[0.089, 0.133]	0.056	[0.014, 0.095]
	Frontoparietal network	0.106	[0.083, 0.129]	0.066	[0.034, 0.106]
		Emotion vs. neutral face (baseline)		Emotion vs. neutral face (2-year)	
voxel	Amygdala	0.013	[−0.017, 0.045]	−0.015	[−0.055, 0.023]
	Hippocampus	−0.015	[−0.040, 0.006]	0.005	[−0.038, 0.043]
	Dorsal attention network	−0.010	[−0.040, 0.016]	0.004	[−0.029, 0.040]
	Ventral attention network	−0.007	[−0.037, 0.018]	0.057	[0.026, 0.090]
	Frontoparietal network	−0.020	[−0.039, 0.000]	0.000	[−0.029, 0.037]
PHATE	Amygdala	−0.006	[−0.033, 0.019]	−0.011	[−0.040, 0.013]
	Hippocampus	−0.019	[−0.044, 0.011]	−0.019	[−0.057, 0.015]
	Dorsal attention network	0.008	[−0.009, 0.027]	0.006	[−0.032, 0.038]
	Ventral attention network	−0.047	[−0.076, −0.018]	−0.005	[−0.037, 0.031]
	Frontoparietal network	−0.005	[−0.037, 0.024]	−0.011	[−0.039, 0.022]
EPHATE	Amygdala	0.083	[0.059, 0.108]	0.057	[0.010, 0.103]
	Hippocampus	0.124	[0.102, 0.133]	0.037	[0.000, 0.075]
	Dorsal attention network	0.087	[0.067, 0.112]	0.063	[0.035, 0.092]
	Ventral attention network	0.111	[0.084, 0.132]	0.057	[0.021, 0.098]
	Frontoparietal network	0.089	[0.063, 0.113]	0.044	[0.010, 0.073]

## Data Availability

The ABCD Study data repository is longitudinal and changes over time and can be found here: nda.nih.gov/abcd. All code specific to this project, including EPHATE software and analysis scripts, is available upon reasonable request and will be released upon publication.
