# Manifold learning uncovers nonlinear interactions between the adolescent brain and social environment that predict psychopathology

**DOI:** 10.1101/2024.02.29.582854

**Published:** 2024-04-04

**Authors:** Erica L. Busch, May I. Conley, Arielle Baskin-Sommers

**Affiliations:** 1 Yale University, Department of Psychology, New Haven, CT, USA

**Keywords:** manifold learning, social environment, brain function, psychopathology, adolescent, mental health

## Abstract

**Background:**

Advanced statistical methods to model the interplay between adolescents and their social environments are essential for understanding how differences in brain function contribute to psychopathology. To progress adolescent mental health research beyond our present achievements – a complex account of brain and environmental risk factors without understanding the neurobiological embedding of the social environment – we need methods to unveil relationships between the developing brain and real-world environmental experiences.

**Methods:**

Here, we investigated associations among psychopathology, social environments, and brain function using participants from the Adolescent Brain and Cognitive Development Study (N=5,235; 2,672 female). Manifold learning is a promising technique for uncovering latent structure from high-dimensional biomedical data like functional magnetic resonance imaging (fMRI). To model brain–social environment interactions and psychopathology, we developed a manifold learning technique called *exogenous* PHATE (E-PHATE). We used E-PHATE embeddings of participants’ brain activation during emotional and cognitive processing to predict measures of cognition and psychopathology both cross-sectionally and longitudinally.

**Results:**

Manifold embeddings of brain activation highlight individual differences in cognition and in psychopathology symptoms which are obscured in high-dimensional (voxel-wise) activity. Specifically, E-PHATE embeddings of participants’ brain activation and social environments at baseline relate to overall psychopathology, externalizing, and internalizing behaviors at both the baseline and at a 2-year follow-up.

**Conclusions:**

Our findings indicate that the adolescent brain’s embedding in the social environment yields enriched insight into psychopathology. Using E-PHATE, we demonstrate how the harmonization of cutting-edge computational methods with longstanding developmental theories advances detection and prediction of adolescent psychopathology.

## Introduction

Nearly 75% of all mental health disorders onset during adolescence, with half of all mental health disorders occurring by age 14^1^. Adolescents who experience mental health problems are at heightened risk for lifelong challenges including lower educational attainment, increased legal system involvement, and chronic physical and psychopathology^2^. Given the sweeping impacts of mental health disorders on individuals and society, developmental scientists have long grappled with understanding the emergence of psychopathology in youth.

Theories and empirical research propose a variety of factors tied to the development of psychopathology^3,4^. However, much of this work has been siloed into research either specifying the role of the social environment or the role of neurobiology in the development of psychopathology. For example, convergent findings suggest that three key brain regions are especially sensitive during adolescent development: prefrontal cortex (PFC), amygdala, and hippocampus^5^. These brain regions support self-regulation and affective processing^6,7^ and differences in the functional activation of these regions have been related various aspects of psychopathology^5^. However, recent studies have called into question the reliability and clinical utility of associations between neuroimaging phenotypes and psychopathology in adolescents^8–10^. When considering brain—behavioral associations, most studies neglect adolescents’ experiences in the social environment^11,12^, which, by themselves, can be risk factors for psychopathology^13^. Meta-analyses report medium to large effects between adversity in adolescents’ families (e.g., conflict, caregiver nonacceptance) and neighborhoods (e.g., experiencing violence or disadvantage) and psychopathology^14–16^. Yet, there is substantial variability in how adolescents respond to their environments, and, increasingly, researchers are examining the complex interactions between adolescent neurobiology and the social environment.

The interaction between adversity in adolescents’ social environment and brain function is broadly supported by the few recent studies testing these components simultaneously. For instance, a recent study found that the interaction of neighborhood adversity and lower executive network activation during an emotional working memory task was related to higher externalizing problems in adolescents^17^. Additional work found that the interaction between neighborhood adversity and decreased amygdala activation during an emotional introspection task was related to higher externalizing problems in a sample of Mexican-origin adolescents^18^. Furthermore, another study found that neighborhood and family adversity interacts with PFC functional connectivity to predict internalizing symptoms^19^. Across these studies, we can stitch together a conceptual model of psychopathology that includes interactions between experiences in adolescent’s social environments and brain function in regions involved in emotional and behavioral regulation. Yet, most previous research has modeled this interaction as a linear combination between univariate measures of brain and environment—in other words, considering a single measurement of social environment and a univariate signal of brain activation. In reality, multidimensional factors shape adolescents’ developing brains and the social environments in which these brains are embedded.

Studying the nonlinear, multidimensional interplay between adolescent brains and environments related to psychopathology risk requires computational methods to combine and unveil structure in high-dimensional, multi-modal data. Manifold learning, a nonlinear approach to dimensionality reduction, is increasingly popular for highlighting complex latent structure in high-dimensional biological data^20^. The algorithm PHATE was specifically designed for high-dimensional, noisy biomedical data and has been applied to uncover local and global latent structure in functional magnetic resonance imaging (fMRI) data^21–23^. Prior research has shown that combining PHATE with additional data (e.g., temporal dynamics of brain responses) enhances the relevance of embeddings for understanding complex cognitive processing (i.e., during movie viewing)^24^. It remains unclear whether PHATE can be 1) used to enhance the behavioral relevance of task-based, developmental fMRI data and 2) combined with environmental data to discover latent geometric structure connecting adolescents’ brains, social environments, and psychopathology.

Here, leveraging the Adolescent Brain Cognitive Development^SM^ Study (ABCD Study^®^) baseline sample and longitudinal data, we investigated the interplay of social environment and brain function on psychopathology. First, we showed that low-dimensional PHATE embeddings of participants’ brain activation during cognitive and emotion processing^25^ were strongly associated with individual differences in working memory performance in 9—10 year-olds. Next, we combined the PHATE brain activation manifold with measurements of adolescents’ social environments into a *multi-view* manifold. Multi-view approaches combine different measurements collected from the same samples into a single representation to be embedded in lower dimensions. For instance, temporal PHATE (T-PHATE) is a recently-introduced multi-view algorithm combining two signals *endogenous* to brain data (i.e., calculated directly from the fMRI measurements). In the present study, we introduce *exogenous* PHATE (E-PHATE), which combines participants’ PHATE brain activation manifold with data about the same participants collected *externally* (i.e., metrics of family and neighborhood adversity). E-PHATE embeddings showed a stronger relationship with psychopathology both cross-sectionally and longitudinally (i.e., two years later, when youth were ages 11–12) than either the PHATE or original brain data^3,4,26,27^. As such, E-PHATE sheds light on the interplay between social environments and activation in several brain regions and improves the detection and prediction of psychopathology in adolescents.

## Methods and materials

### Participants

Participants were adolescents included in ABCD Data Release 4.0 (DOI:10.15154/1523041). Neuroimaging and environmental data were from the baseline assessment (ages 9–10). Psychopathology data were from the baseline and the 2-year follow up (ages 11–12). Participants from the ABCD Study dataset were excluded if they had missing fMRI, environmental, or mental health measures, resulting in 5,235 participants included in baseline analyses. Of those, 2,686 participants had complete data at the two-year follow up. Information about the ABCD study and our sample demographics are detailed in Table 1 and Supplemental Methods.

### Social environment measures

Measures of the social environment were selected to characterize youth’s family and neighborhood social environments at the baseline timepoint. Family environment was measured using participants’ perception of family threat (anger and conflict expressed among family members) and of family support (caregiver acceptance). Neighborhood environment was measured using participant and caregiver assessment of perceived safety/crime, and the Area Deprivation Index (ADI), a composite index of neighborhood socioeconomic disadvantage. More details about social environment measures are included in Supplemental Methods.

### Psychopathology measures

Measures of adolescent psychopathology were assessed using *t*-scores from the baseline and two-year follow-up data from the Achenbach System of Empirically Based Assessment Child Behavior Checklist (CBCL), which is a 119 item parent/caregiver-report survey of adolescent psychopathology validated for use in children ages 6–18^28^. Primary analyses examined total problems and externalizing and internalizing broad-band scales. Supplemental analyses examined anxious/depression, withdrawn/depression, aggression, and rule-breaking behavior syndrome scales.

### Emotional n-back task

The in-scanner emotional n-back (EN-back) task was designed to engage emotion and memory processing^25,29^. The task included two ~5-minute fMRI runs, each with eight task blocks and four 15s fixation blocks. During each run, participants performed four 0-back (low memory load) and four 2-back (high memory load) blocks with happy, fear, or neutral face or place stimuli. For each trial, stimuli were presented for 2s and followed by 500ms fixation cross. On 0-back blocks, participants were instructed to press “match” when the stimulus was the same as a target stimulus presented at the beginning of the block, and “no match” if not. On 2-back blocks, participants were instructed to press “match” when the stimulus was the same as the stimulus presented two trials back, and “no match” if not. EN-back behavioral performance was measured with sensitivity, calculated as d’ = z(hits) - z(false alarms) and adjusted for extreme values^30^.

### Neuroimaging data

All fMRI data were preprocessed by the ABCD Study Data Analysis, Informatics, and Resource Center (DAIRC) as detailed elsewhere^31^. Task, acquisition, and processing information is included in Supplemental Methods. Following preprocessing, EN-back activation was estimated for each participant using general linear models with fixation, 2-back and 0-back condition, and happy, fearful, and neutral face and place stimuli as predictors^45^. We evaluated emotion processing activation with the linear contrast of emotional and neutral face task blocks and cognitive processing activation with the linear contrast of 2-back and 0-back task blocks, as in prior studies^17,32^. For the two EN-back contrasts (2-back vs. 0-back and emotion face vs. neutral), we analyzed beta weights from networks defined using the Schaefer parcellation^33^, which delineates 400 cortical parcels within 7 networks^34^, and the Scale I Tian subcortical parcellation^35^, which delineates 8 bilateral subcortical regions. Multivoxel activation patterns were extracted from three networks (frontoparietal, dorsal attention, and ventral attention networks) and two bi-lateral subcortical structures (amygdala and hippocampus). Multivariate activation patterns were then vectorized, resulting in one vector for each region, participant, and contrast.

### Manifold embeddings

Manifold learning was used to test whether a low-dimensional embedding of participants’ brain activation patterns better captured behaviorally-relevant activation during cognitive and emotion processing. Specifically, we applied the PHATE algorithm, a diffusion-based manifold learning method developed for performing dimensionality reduction on high-throughput biomedical data**.** PHATE captures local neighborhoods in the data using a diffused Markov transition matrix and maintains global data context using a potential distance metric^21^. This allows PHATE manifolds to reflect local and global manifold structure and perform effective denoising and makes PHATE well suited for the high dimensionality and intrinsic noise of fMRI activity. Despite the low reliability^36^ and aforementioned challenges of developmental task-based neuroimaging data, we suspected that individual differences in brain function would be highlighted in low-dimensional PHATE manifold and would, in turn, better relate to cognition and mental health relative to voxel data^22,24^.

To address our hypothesis – that the way adolescent social environments and brain function relates to psychopathology is complex and multifaceted, but can be characterized by a low-dimensional, nonlinear manifold – we designed the *exogenous* PHATE algorithm (E-PHATE). In previous work, we found that fMRI timeseries data was best captured using a dual-diffusion process to combine two *endogenous*, interacting signals present in neuroimaging data: activation across space and activation which unfurls over time^24^. In the current study, we applied a similar dual-diffusion approach to combine *exogenous* information about adolescents’ social environments with their brain activation. The *exogenous* PHATE (E-PHATE) procedure combines the PHATE diffusion matrix and a second view based on an affinity matrix over participants’ scores on additional, external variables. We built the exogenous factor view of E-PHATE to capture individual differences in participants’ scores across five metrics: family conflict, caregiver acceptance, youth and caregiver perceived neighborhood crime/safety, and neighborhood disadvantage (the Area Deprivation Index). Further information about these measures and the E-PHATE algorithm are outlined in Supplemental Methods. For all analyses, PHATE and E-PHATE embeddings were 20 dimensional, which was selected based on prior literature^22,24^. Embeddings were performed separately for each region/network and task contrast.

### Associations with task performance and psychopathology outcomes

We used a linear regression with 20-fold cross-validation measure associations between task performance or psychopathology outcomes and neuroimaging data (either voxel resolution ROI data their embeddings with either PHATE or E-PHATE). Cross-sectional association used participants’ brain data at baseline to predict CBCL scores at the baseline time point. Longitudinal prediction used participants’ brain data at baseline to predict their psychopathology scores at the 2-year follow-up. Longitudinal prediction only trained on participants who had both baseline and 2-year follow up data (*n*=2,686) whereas the cross-sectional association included participants with complete baseline data (*n*=5,235). Model performance was evaluated as prediction accuracy on held-out participants, which was then averaged across the 20 cross-validation folds. P-values comparing the performance of regression models trained on different data representations were obtained using permutation tests (10,000 iterations) with Bonferroni corrected, performed within contrast but across the 5 ROIs. Further details about model assessment are included in Supplemental Methods.

Analysis code and software for E-PHATE will be released upon publication.

## Results

### PHATE strengthened associations between brain activation and task performance

**Figure 2:**
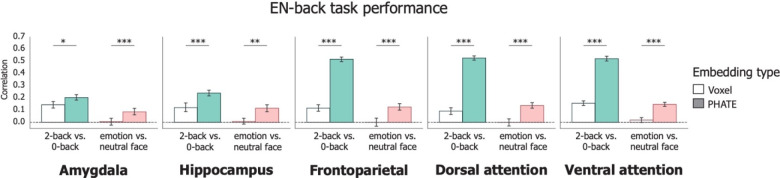
Associations of brain data with EN-back task performance Bars represent average correlation between model predicted and true EN-back scores on held-out participants’ data. Left column in each graph represents the 2-back vs. 0-back contrast; Right column in each graph represents the emotion vs. neutral contrast. Error bars represent the 95% confidence interval of the mean across 20 cross-validation folds. ~ p < 0.1, * p < 0.05, ** p < 0.01, *** p < 0.001

To confirm the utility of manifold learning for highlighting cognitively-relevant brain activity, we first validated that standard PHATE embeddings of brain activation from regions implicated in emotion and memory processing enhanced prediction of EN-back task performance relative to the full voxel-dimensional data. In all regions evaluated, regression models trained on the PHATE embeddings significantly outperformed those trained on the voxel data (Figure 2, Table 2; Supplemental Data 2). PHATE embeddings from the 2-back vs. 0-back contrast were significantly related to EN-back task performance in all regions, with the largest magnitude of effects (*r* > .50) in the frontoparietal and attention networks. In comparison, voxel data in response to the 2-back vs. 0-back contrast showed smaller (*r* < 0.20) associations with EN-back performance in all regions (Table 2). Given previous research linking frontoparietal and attention networks with higher order cognitive abilities and working memory^32,37,38^, these results demonstrate that PHATE optimized the utility of fMRI data for detecting behaviorally relevant brain activation during cognitive processing.

PHATE embeddings from the emotion vs. neutral contrast showed moderate associations with EN-back performance (*r >* 0.12 in frontoparietal and attention networks). In contrast, and consistent with prior research^32^, none of the voxel data for the emotion vs. neutral face contrast significantly related to EN-back performance. Therefore, embedding brain activation with PHATE enhanced the relevance of emotion processing-related brain activation for understanding performance during the EN-back.

### E-PHATE enhanced cross-sectional associations with psychopathology

Standard PHATE implementations cannot account for interactions of additional variables outside of the high-dimensional input data (i.e., brain activation). However, outside factors, such as the social environment, also can inform the individual variation in neurobiology associated with psychopathology. We tested whether E-PHATE strengthened associations between brain function and psychopathology symptoms at baseline, using scores from the Child Behavior Checklist (CBCL)^28^.

**Figure 3:**
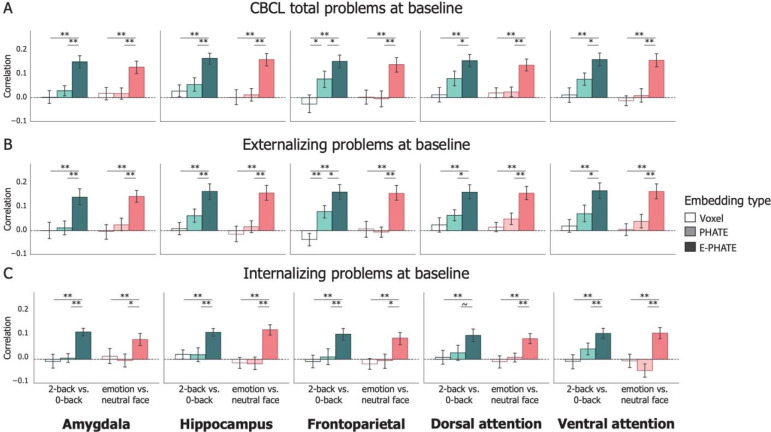
Cross-sectional associations of brain data with mental health problems Bars represent average correlation between model predicted and true mental health problem scores on held-out participants’ data, trained and tested with 20-fold cross validation. Both mental health scores and brain/environmental data were collected at the baseline timepoint. Left column in each graph represents the 2-back vs. 0-back contrast; Right column in each graph represents the emotion vs. neutral contrast. A=Total problems; B=Externalizing problems; C=Internalizing problems. Error bars represent the 95% confidence interval of the mean across 20 cross-validation folds. ~ p < 0.1, * p < 0.05, ** p < 0.01, *** p < 0.001

Voxel activity from the 2-back vs. 0-back working memory contrast significantly related to the CBCL total problem score above chance in only the hippocampus (Table 2). PHATE embeddings of the 2-back vs. 0-back contrast were significantly related to total problem scores greater than chance in all ROI. However, they only outperformed voxel data significantly in the frontoparietal network. E-PHATE reflected stronger associations between brain activation and total problem scores relative to both the voxel data and PHATE embeddings for the 2-back vs. 0-back contrast for every region (all p’s < 0.05, corrected). Magnitude of associations between E-PHATE embeddings and overall psychopathology were similarly high across all regions (Figure 3A; Table 2; Supplemental Data 2).

Replicating previous research showing null associations between emotion-processing activation and psychopathology^39^, voxel activity from the emotion vs. neutral contrast did not significantly relate to individual differences in CBCL total problem scores in any ROI. Without added information about the social environment, PHATE embeddings of the emotion vs. neutral contrast performed similarly to the voxel data in the strength of its relationship with total problem scores. E-PHATE significantly enhanced this relationship relative to the voxel data and PHATE embeddings in the emotion vs. neutral contrasts for all regions (all p’s < 0.01, corrected). The magnitude of E-PHATE’s performance with emotion vs. neutral contrast was comparably strong to E-PHATE’s performance with the 2-back vs. 0-back contrast (Figure 3A; Table 2; Supplemental Data 2).

The CBCL total problem score can be further broken into two main broad-band scales: externalizing and internalizing problems. To examine whether the significant relationships between E-PHATE embeddings and total problems were driven by associations with externalizing or internalizing problems specifically, we repeated the analyses above to predict t-scores for each of the broad-band CBCL scales independently. As for the overall scores, E-PHATE embeddings were more strongly related to externalizing problems relative to the voxel data or PHATE embeddings across both task contrasts in all ROIs (all p’s < 0.05; Figure 3B; Table 2). The magnitude of effects for internalizing problems was generally lower than that of externalizing problems yet still significant relative to chance, which was not the case for voxel or PHATE embeddings. (Figure 3C; Table 2).

Supplementary analyses replicated this pattern across the externalizing and internalizing subscale symptoms too: E-PHATE embeddings were significantly associated with all subscale symptoms, with the strength of the association depending upon the subscale, fMRI contrast, and ROI. As with the broad-band scales, E-PHATE embeddings more strongly reflected externalizing symptoms (aggression, rule-breaking) than they do internalizing symptoms (anxious/depressed, withdrawn/depressed, somatization) (Supplementary Figure 1; Supplemental Data 1).

To confirm that the increased sensitivity of E-PHATE for detecting psychopathology was attributable to added information specifically about the social environment, as opposed to an increase in the sheer quantity of data about each participant, we replaced the social environment factor matrix with other exogenous information about participants that should be irrelevant to psychopathology, including height, weight, handedness, number of siblings, and age (in months). Adding irrelevant information to the E-PHATE manifold hindered prediction of psychopathology. Thus, this sensitivity analysis confirmed that the quantity of information in E-PHATE did not drive its enhanced behavioral relevance (Supplementary Figure 2; Supplemental Data 2).

In addition, we asked whether specific variables about the social environment primarily drove the sensitivity of E-PHATE to psychopathology. We considered second views of the E-PHATE matrix built solely upon neighborhood disadvantage (ADI) and family conflict, which are commonly considered in isolation as measures of environmental adversity^40,41^. While adding either neighborhood disadvantage or family conflict alone improved associations with psychopathology relative to no environmental information (voxel or PHATE data), neither variable in isolation afforded as great an improvement as the five-feature social environment view (Supplementary Figure 3).

### E-PHATE improved longitudinal prediction of psychopathology

To evaluate whether the signals highlighted by E-PHATE could enhance our ability to detect brain activation relevant for future psychopathology, we asked whether the same latent space built from brain and social environmental factors at baseline (ages 9–10) could predict psychopathology two years later (at ages 11–12). Using a subset of the original participants (*n* = 2,686 with complete 2-year follow-up data), we embedded baseline brain activation data and social environment factors with E-PHATE and used linear regression to predict CBCL total, externalizing, and internalizing problems at the two-year follow-up.

**Figure 4:**
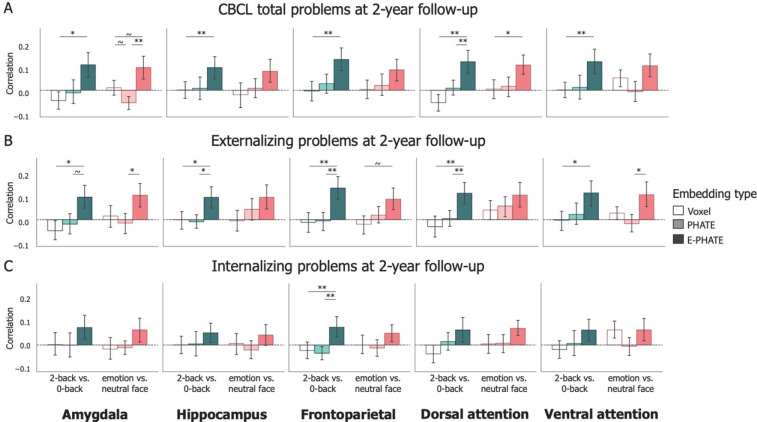
Longitudinal prediction of psychopathology Bars represent average correlation between model predicted and true problem scores on held-out participants’ data, trained and tested with 20-fold cross validation. Brain and environment data were collected from the baseline timepoint, and psychopathology scores were collected at the 2-year follow-up. Left column in each graph represents the 2-back vs. 0-back contrast; Right column in each graph represents the emotion vs. neutral contrast. A=Total problems; B=Externalizing problems; C=Internalizing problems. Error bars represent the 95% confidence interval of the mean across 20 cross-validation folds. ~ p < 0.1, * p < 0.05, ** p < 0.01, *** p < 0.001

E-PHATE embeddings for several contrasts and regions significantly predicted future psychopathology (Table 2; Supplemental Data 1). Specifically, E-PHATE embeddings from the 2-back vs. 0-back contrast better predicted total problem and externalizing scores from hippocampus and amygdala (p’s < 0.05, corrected) and frontoparietal and attention network activation (p’s < 0.01, corrected) relative to voxel resolution data. E-PHATE embeddings from the 2-back vs. 0-back contrast also predicted total problem and externalizing scores from dorsal attention network activation and predicted externalizing scores from frontoparietal network activation significantly better than PHATE embeddings (Figure 4A; Supplemental Data 2). The effect sizes for longitudinal prediction were generally lower for all regions and contrasts relative to the cross-sectional results.

As with the cross-sectional analysis, longitudinal prediction of internalizing scores was lower compared to total problem or externalizing scores (Figure 4B). Internalizing problems were only significantly predicted by E-PHATE embeddings from the 2-back vs. 0-back contrast of frontoparietal network activation (Figure 4C; Table 2; Supplemental Data 1).

Consistent with previous research linking aberrant amygdala function during emotion processing with psychopathology^42,43^, E-PHATE embeddings of amygdala activation from the emotion vs. neutral contrast predicted total and externalizing problems better than voxel or PHATE embeddings. E-PHATE embeddings from the emotion vs. neutral contrast also predicted externalizing problems from dorsal attention activation (Figure 4B; Table 2). Overall, by including information about participants’ social environments at ages 9—10 with their brain activation manifolds, we gain insight into developing symptoms of psychopathology two years later.

## Discussion

Decades of theories and empirical research indicate that both an adolescent’s neurobiology and social environment interact in the development of psychopathology. Yet, prior work has struggled to represent the complexity of this interplay; measurements of adolescent neurobiology and psychopathology are noisy and frequently unreliable^36,44^, and the embedding of the adolescent brain into its environment is multi-dimensional and nonlinear. Here, we address these limitations using nonlinear manifold learning, a type of dimensionality reduction used for cleaning and uncovering latent structure in noisy, high-dimensional biomedical data^20^. We found that manifold learning using PHATE^21^ enhanced the relevance of task-based fMRI data for understanding task performance and mental health problems in a large, sociodemographic diverse sample of US adolescents. Across multiple brain regions and cortical networks implicated in cognitive and emotion processing, PHATE uncovered structure in the brain activation data which was significantly associated with task performance, but likely obscured high-dimensionality and noise at the voxel level.

Taken alone, though, standard PHATE embeddings did not improve insight into psychopathology; they were more associated with overall psychopathology and externalizing problems than the voxel data only in the frontoparietal network. However, theoretical models and past empirical work indicates that other regions and networks, such as the amygdala and hippocampus, are implicated in psychopathology^17,43,45,46^. Manifold learning using E-PHATE, our new multi-view approach to integrate brain activation data with exogenous measures, vastly improved detection and prediction of psychopathology. Overall, our results demonstrate that manifold learning techniques are well-suited for the complexity of multimodal developmental data and have great potential to enhance research on the neurobiology of psychopathology in adolescents.

A major goal of developmental science is to represent the interplay between youth and their broader social environments in order to identify early markers of risk and novel targets for intervention^3,4,17,18,26,27^. This work offers a substantial methodological advance toward that goal through the development of E-PHATE. First, E-PHATE offers researchers an data-driven method for capturing the nonlinear interactions of biological—environmental factors, as opposed linear approaches which view interactions as a simple product of two variables^47^. Second, by incorporating exogenous information about adolescents’ neighborhoods and families as essential data lending structure to the brain activation manifold, E-PHATE improved associations between brain function and psychopathology. Previous studies have questioned the reliability of empirical support linking specific ROIs (e.g., amygdala) to psychopathology in youth^8–10^, yet E-PHATE highlighted signals relevant for understanding psychopathology in every ROI and network we examined across both contrasts. These results demonstrate that efforts to elucidate relationships between adolescent brain function and psychopathology may be stifled if researchers fail to consider the broader context in which brain development is embedded^27,48,49^.

Beyond the applications in developmental neuroscience and clinical psychology outlined above, E-PHATE shows promise for a variety of big-data challenges. E-PHATE addresses a key limitation of many manifold learning methods, which identify latent structure in an unsupervised fashion. This means the algorithms cannot integrate or evaluate the interplay of multiple measurement types into one latent structure. E-PHATE affords combining different measurements of the same samples into the manifold calculation and maintains both the benefits of unsupervised manifold geometry discovery and hypothesized structure.

The present work should be viewed in light of a few limitations. First, we focused on specific ROIs and networks that have previously been related to memory- and emotion-processing and mental health^5,6,17,25,32,37,38^. Yet, investigating other brain areas or whole-brain approaches may be relevant for understanding task performance and psychopathology. Second, manifold learning algorithms are not able to discern the direction or specific patterns of brain activation that contribute to associations with task performance and psychopathology. Third, while E-PHATE could predict psychopathology two years later, results from the current study are correlational and cannot speak to causation. Considering research showing bidirectional relationships between the social environment and psychopathology^50,51^, future studies should incorporate manifold learning within other longitudinal designs. Fourth, the results in the current paper only reflect a snapshot of development. Since the peak onset of psychopathology is later in adolescence^1^, future research investigating a larger developmental window is needed.

In all, we present evidence for a complex interplay between social environments, brain function, and psychopathology as uncovered by a new, general-purpose method with interdisciplinary applications. Ultimately, data-driven, interdisciplinary approaches that characterize adolescents’ changing neurobiology within the context of their social environments may allow us to identify early markers of risk and novel targets for intervention.

## Supplementary Material

Supplement 1

## Figures and Tables

**Figure 1:**
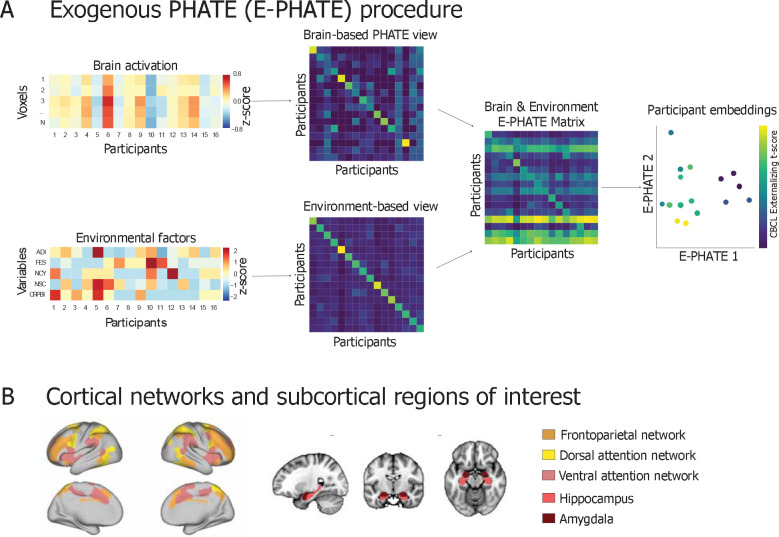
Exogenous PHATE (E-PHATE) procedure **A:** E-PHATE models the interactions between brain activation and exogenous information about participants using multi-view manifold learning. Here, the first view of E-PHATE takes as inputs a vector of voxel-wise beta values from a given region of interest for each participant in the sample and learns a PHATE-based affinity matrix between each participants’ brain activations. The second view takes a vector of social environment scores for each of those participants and builds an affinity matrix across those scores, which is then row-normalized to become a transition probability matrix. These two views are then combined into one matrix (the E-PHATE diffusion operator) which jointly characterizes both the brain and environment manifold geometry. This matrix is then embedded into lower dimensions using metric multidimensional scaling (m-MDS). Participants’ coordinates in E-PHATE dimensions visually reflect individual differences along psychopathology scores (here, externalizing problem scores). Main analyses presented here include 5 factors of social environment: ADI = area deprivation index, a composite index of neighborhood disadvantage; FES = family conflict; NCY = youth perceived neighborhood safety/crime; NSC = caregiver perceived neighborhood safety/crime; CRPBI = caregiver acceptance **B:** Subsequent analyses are presented using beta values extracted for voxels in the bilateral amygdala and hippocampus and surface vertices in three cortical networks: frontoparietal, dorsal and ventral attention networks.

**Table 1.** Demographics for participants included in the baseline (ages 9–10) and 2-year follow-up (ages 11–12) analyses.

Timepoint	Baseline	2-year follow-up
**n (percent)**	5,235	2,686
**Female**	2,672 (51)	1,338 (50)
**Race/Ethnicity**		
Hispanic or Latinx	942 (18.0)	477 (17.8)
Black	535 (10.2)	240 (8.9)
White	3,076 (58.8)	1,654 (61.6)
Asian	113 (2.2)	52 (1.9)
Other	569 (10.9)	263 (9.8)
**Caregiver education (%)**		
< HS Diploma	160 (3)	77 (2.9)
HS Diploma / GED	366 (6.9)	156 (5.8)
Some College	1,194 (22.8)	628 (23.4)
Bachelor	1,422 (27.2)	730 (27.2)
Post Graduate Degree	2,090 (39.9)	1,091 (40.6)
Unknown	3 (0.05)	4 (0.1)
**Family income (%)**		
< $50K	1,116 (21.3)	566 (21.1)
> $50K - < $100K	1,435 (27.4)	1,166 (43.4)
> $100K	2,333 (44.6)	786 (29.3)
Unknown	351 (6.4)	168 (6.2)

**Table 2.** Results showing associations between brain activation embeddings and task performance and psychopathology.

Embedding type	ROI	Pearson’s r (baseline)	95% CI (baseline)	Pearson’s r (2-year)	95% CI (2-year)
**EN-Back performance**					
2-back vs. 0-back					
voxel	Amygdala	0.145	[0.116, 0.172]	n/a	
	Hippocampus	0.121	[0.088, 0.164]		
	Dorsal attention network	0.093	[0.066, 0.177]		
	Ventral attention network	0.157	[0.088, 0.164]		
	Frontoparietal network	0.117	[0.090, 0.144]		
PHATE	Amygdala	0.203	[0.182, 0.228]		
	Hippocampus	0.241	[0.219, 0.265]		
	Dorsal attention network	0.528	[0.511, 0.546]		
	Ventral attention network	0.523	[0.504, 0.541]		
	Frontoparietal network	0.519	[0.498, 0.537]		
Emotion vs. neutral face					
voxel	Amygdala	0.007	[−0.032, 0.029]	n/a	
	Hippocampus	0.008	[−0.013, 0.031]		
	Dorsal attention network	0.001	[−0.030, 0.026]		
	Ventral attention network	0.019	[−0.002, 0.038]		
	Frontoparietal network	0.001	[−0.029, 0.036]		
PHATE	Amygdala	0.087	[0.063, 0.113]		
	Hippocampus	0.117	[0.089, 0.145]		
	Dorsal attention network	0.138	[0.118, 0.165]		
	Ventral attention network	0.147	[0.130, 0.164]		
	Frontoparietal network	0.127	[0.102, 0.156]		
**CBCL total problems**					
		2-back vs. 0-back (baseline)		2-back vs. 0-back (2-year)	
voxel	Amygdala	0.002	[−0.030, 0.027]	−0.039	[−0.070, −0.006]
	Hippocampus	0.028	[0.004, 0.051]	0.001	[−0.028, 0.036]
	Dorsal attention network	0.012	[−0.016, 0.044]	−0.046	[−0.078, −0.018]
	Ventral attention network	0.011	[−0.021, 0.040]	0.001	[−0.026, 0.033]
	Frontoparietal network	−0.027	[−0.062, 0.011]	−0.003	[−0.037, 0.038]
PHATE	Amygdala	0.029	[0.007, 0.049]	−0.010	[−0.047, 0.040]
	Hippocampus	0.055	[0.030, 0.083]	0.008	[−0.031, 0.051]
	Dorsal attention network	0.081	[0.047, 0.107]	0.008	[0.047, 0.107]
	Ventral attention network	0.078	[0.052, 0.104]	0.011	[−0.018, 0.043]
	Frontoparietal network	0.078	[0.046, 0.108]	0.024	[−0.021, 0.057]
E-PHATE	Amygdala	0.150	[0.125, 0.175]	0.095	[0.054, 0.146]
	Hippocampus	0.165	[0.141, 0.187]	0.085	[0.044, 0.123]
	Dorsal attention network	0.155	[0.131, 0.182]	0.106	[0.066, 0.158]
	Ventral attention network	0.160	[0.135, 0.188]	0.107	[0.061, 0.151]
	Frontoparietal network	0.153	[0.126, 0.179]	0.115	[0.077, 0.163]
		Emotion vs. neutral face (baseline)		Emotion vs. neutral face (2-year)	
voxel	Amygdala	0.018	[−0.007, 0.043]	0.009	[−0.013, 0.038]
	Hippocampus	0.001	[−0.029, 0.034]	−0.017	[−0.069, 0.025]
	Dorsal attention network	0.020	[0.000, 0.042]	0.005	[−0.030, 0.034]
	Ventral attention network	−0.013	[−0.033, 0.006]	0.046	[0.014, 0.075]
	Frontoparietal network	0.003	[−0.026, 0.032]	0.003	[−0.029, 0.038]
PHATE	Amygdala	0.017	[−0.006, 0.043]	−0.047	[−0.074, −0.026]
	Hippocampus	0.012	[−0.018, 0.039]	0.007	[−0.027, 0.040]
	Dorsal attention network	0.024	[0.003, 0.043]	0.015	[−0.026, 0.046]
	Ventral attention network	0.009	[−0.020, 0.041]	−0.005	[−0.045, 0.029
	Frontoparietal network	−0.005	[−0.036, 0.027]	0.018	[−0.017, 0.065]
E-PHATE	Amygdala	0.129	[0.104, 0.154]	0.085	[0.044, 0.129]
	Hippocampus	0.160	[0.135, 0.186]	0.071	[0.035, 0.121]
	Dorsal attention network	0.137	[0.113, 0.162]	0.094	[0.062, 0.134]
	Ventral attention network	0.157	[0.130, 0.184]	0.091	[0.056, 0.146]
	Frontoparietal network	0.139	[0.110, 0.169]	0.076	[0.031, 0.115]
**CBCL externalizing problems**					
		2-back vs. 0-back (baseline)		2-back vs. 0-back (2-year)	
voxel	Amygdala	0.001	[−0.040, 0.032]	−0.042	[−0.073, −0.002]
	Hippocampus	0.009	[−0.017, 0.032]	−0.001	[−0.037, 0.034]
	Dorsal attention network	0.024	[−0.007, 0.053	−0.027	[−0.062, 0.019]
	Ventral attention network	0.020	[−0.009, 0.043]	−0.003	[−0.036, 0.034]
	Frontoparietal network	−0.037	[−0.059, −0.011]	−0.010	[−0.050, 0.028]
PHATE	Amygdala	0.012	[−0.014, 0.041]	−0.017	[−0.051, 0.020]
	Hippocampus	0.062	[0.034, 0.089]	−0.008	[−0.032, 0.022]
	Dorsal attention network	0.064	[0.039, 0.089]	0.004	[−0.024, 0.037
	Ventral attention network	0.071	[0.031, 0.104]	0.020	[−0.017, 0.061]
	Frontoparietal network	0.080	[0.053, 0.106]	−0.005	[−0.046, 0.025]
E-PHATE	Amygdala	0.140	[0.108, 0.175]	0.084	[0.045, 0.130]
	Hippocampus	0.165	[0.131, 0.196]	0.084	[0.048, 0.124]
	Dorsal attention network	0.162	[0.133, 0.192]	0.099	[0.058, 0.144]
	Ventral attention network	0.167	[0.135, 0.201]	0.100	[0.047, 0.138]
	Frontoparietal network	0.162	[0.133, 0.195]	0.118	[0.075, 0.159]
		Emotion vs. neutral face (baseline)		Emotion vs. neutral face (2-year)	
voxel	Amygdala	−0.003	[−0.036, 0.024]	0.013	[−0.024, 0.051]
	Hippocampus	−0.015	[−0.046, 0.018]	−0.004	[−0.042, 0.037]
	Dorsal attention network	0.014	[−0.005, 0.033]	0.036	[0.000, 0.071]
	Ventral attention network	0.005	[−0.023, 0.027]	0.024	[0.000, 0.047]
	Frontoparietal network	0.007	[−0.028, 0.038]	−0.017	[−0.052, 0.013]
PHATE	Amygdala	0.025	[0.004, 0.056]	−0.013	[−0.050, 0.023]
	Hippocampus	0.016	[−0.010, 0.042]	0.038	[0.003, 0.079]
	Dorsal attention network	0.049	[0.026, 0.073]	0.051	[0.012, 0.085]
	Ventral attention network	0.038	[0.010, 0.070]	−0.016	[−0.045, 0.021]
	Frontoparietal network	−0.006	[−0.028, 0.016]	0.017	[−0.013, 0.052]
E-PHATE	Amygdala	0.143	[0.121, 0.169]	0.091	[0.049, 0.137]
	Hippocampus	0.158	[0.129, 0.191]	0.084	[0.049, 0.141]
	Dorsal attention network	0.157	[0.132, 0.187]	0.092	[0.045, 0.138]
	Ventral attention network	0.165	[0.129, 0.194]	0.093	[0.048, 0.137]
	Frontoparietal network	0.157	[0.130, 0.191]	0.007	[0.037, 0.118]
**CBCL internalizing problems**					
		2-back vs. 0-back (baseline)		2-back vs. 0-back (2-year)	
voxel	Amygdala	−0.009	[−0.040, 0.019]	0.002	[−0.035, 0.041]
	Hippocampus	0.021	[0.003, 0.040]	0.001	[−0.032, 0.032]
	Dorsal attention network	0.008	[−0.023, 0.037]	−0.033	[−0.064, −0.001]
	Ventral attention network	−0.009	[−0.045, 0.020]	−0.018	[−0.051, 0.013]
	Frontoparietal network	−0.010	[−0.036, 0.017]	−0.021	[−0.048, 0.015]
PHATE	Amygdala	0.005	[−0.012, 0.023]	−0.001	[−0.041, 0.045]
	Hippocampus	0.019	[−0.009, 0.053]	0.004	[−0.035, 0.059]
	Dorsal attention network	0.027	[−0.004, 0.056]	0.013	[−0.020, 0.050]
	Ventral attention network	0.044	[0.018, 0.068]	0.006	[−0.042, 0.047]
	Frontoparietal network	0.010	[−0.020, 0.044]	−0.031	[−0.059, −0.009]
E-PHATE	Amygdala	0.115	[0.100, 0.132]	0.065	[0.026, 0.117]
	Hippocampus	0.113	[0.095, 0.131]	0.045	[0.006, 0.081]
	Dorsal attention network	0.101	[0.077, 0.130]	0.056	[0.010, 0.100]
	Ventral attention network	0.109	[0.089, 0.133]	0.056	[0.014, 0.095]
	Frontoparietal network	0.106	[0.083, 0.129]	0.066	[0.034, 0.106]
		Emotion vs. neutral face (baseline)		Emotion vs. neutral face (2-year)	
voxel	Amygdala	0.013	[−0.017, 0.045]	−0.015	[−0.055, 0.023]
	Hippocampus	−0.015	[−0.040, 0.006]	0.005	[−0.038, 0.043]
	Dorsal attention network	−0.010	[−0.040, 0.016]	0.004	[−0.029, 0.040]
	Ventral attention network	−0.007	[−0.037, 0.018]	0.057	[0.026, 0.090]
	Frontoparietal network	−0.020	[−0.039, 0.000]	0.000	[−0.029, 0.037]
PHATE	Amygdala	−0.006	[−0.033, 0.019]	−0.011	[−0.040, 0.013]
	Hippocampus	−0.019	[−0.044, 0.011]	−0.019	[−0.057, 0.015]
	Dorsal attention network	0.008	[−0.009, 0.027]	0.006	[−0.032, 0.038]
	Ventral attention network	−0.047	[−0.076, −0.018]	−0.005	[−0.037, 0.031]
	Frontoparietal network	−0.005	[−0.037, 0.024]	−0.011	[−0.039, 0.022]
E-PHATE	Amygdala	0.083	[0.059, 0.108]	0.057	[0.010, 0.103]
	Hippocampus	0.124	[0.102, 0.133]	0.037	[0.000, 0.075]
	Dorsal attention network	0.087	[0.067, 0.112]	0.063	[0.035, 0.092]
	Ventral attention network	0.111	[0.084, 0.132]	0.057	[0.021, 0.098]
	Frontoparietal network	0.089	[0.063, 0.113]	0.044	[0.010, 0.073]
