# Supplementary material for: Manifold learning uncovers nonlinear interactions between the adolescent brain and social environment that predict psychopathology
Source: bioRxiv. 2024 Apr 4:2024.02.29.582854. Originally published 2024 Mar 6. Preprint. [Version 2] doi: 10.1101/2024.02.29.582854 (PMC10942356; doi:10.1101/2024.02.29.582854)
Supplement: Supplementary file 1 [file media-1.docx]

**Supplemental materials**

**Supplemental methods**

*ABCD Study Participants*

The ABCD Study is a longitudinal study following youth from 21 sites across the United States^1^. Study procedures received centralized University of California, San Diego and site-specific institutional review board approval^2^. Parents or caregivers provided written informed consent and adolescents gave written assent for study participation. ABCD study-wide exclusion criteria included a diagnosis of schizophrenia, moderate to severe autism spectrum disorder, intellectual disability, substance use disorder at recruitment, persistent major neurological disorders, multiple sclerosis, sickle cell disease, seizure disorders like Lennox-Gastaut Syndrome, Dravet Syndrome, and Landau Kleffner Syndrome.

*Social environment measures*

Measures of the social environment were selected to characterize youth’s family and neighborhood social environments using baseline assessments from the ABCD Study Culture and Environment^3^ and Linked External Data^4^ protocols. 

Family threats were measured with the youth-report ABCD Family Environment Scale–Family Conflict Subscale, which included nine items measuring youth’s perception of anger and conflict expressed among family members. For each item, youth endorsed whether a statement was true or false for most family members. All items were summed with higher scores indicating *more* family conflict. 

Family support and acceptance was assessed with the Children’s Report of Parental Behavioral Inventory, which included five items measuring youth’s perception of caregiver acceptance and support. For each item, youth indicated whether a statement was not, somewhat, or a lot like their primary caregiver. All items were averaged and reverse scored such that higher scores indicated *less* family support/acceptance. 

Neighborhood threats were measured using the ABCD Neighborhood Safety/Crime Survey, which assessed perceived safety/crime in participants’ neighborhoods. Because the adolescent assessment included only a single item asking youth whether they strongly agreed or strongly disagreed with a statement about their safety from neighborhood crime, neighborhood threats were measured as the mean of all adolescent- and caregiver-report items. The caregiver survey included the exact item from the youth survey with two other items asking whether caregivers strongly agreed or strongly disagreed with statements about their perceived safety while walking and violence being a problem in their neighborhood. All adolescent- and caregiver-report items were reverse-scored such that higher scores indicated *more* neighborhood threats. 

Neighborhood deprivation was measured using the Area Deprivation Index (ADI), which is a composite index of neighborhood socioeconomic disadvantage based on income, education, employment and housing quality in a census tract derived from the American Community Survey. Participants’ ADI national percentile were calculated for their primary address, and higher scores indicated *more* neighborhood deprivation. 

*Neuroimaging data*

fMRI data were collected on Siemens Prisma, Phillips, and GE 750 3T scanners using a 32-channel head coil^1^.  Functional images were collected with a multiband gradient echo-planar imaging sequence and the following parameters: TR = 800 ms, TE = 30 ms, flip angle = 52°, 60 slices acquired in the axial plane, voxel size = 2.4mm^3^, multiband slice acceleration factor = 6. All fMRI data were preprocessed by the ABCD Study Data Analysis, Informatics, and Resource Center (DAIRC) as detailed elsewhere^5^. Participants were excluded based on low quality structural scans, fewer than 550 degrees of freedom in preprocessed, concatenated timeseries data, task contrast maps missing over 260 grayordinate values (e.g., missing values outside the medial wall), and extreme values (> 3 SD from the group mean) for the mean or SD of beta weights across all grayordinates, as in prior studies^6^.  

*Exogenous PHATE (E-PHATE) procedure*

Given a matrix http://www.sciweavers.org/tex2img.php?bc=Transparent&fc=Black&im=jpg&fs=100&ff=modern&edit=0&eq=X%20%3D%20x_1%2C%20x_2%2C%20%E2%80%A6%20x_n, where https://www.codecogs.com/eqnedit.php?latex=x_n%20%5Cin%20%7B%7B%5Cmathbb%7BR%7D%7D%7D%5E%7BN%7D for participant https://www.codecogs.com/eqnedit.php?latex=n is a https://www.codecogs.com/eqnedit.php?latex=V-dimensional vector, and https://www.codecogs.com/eqnedit.php?latex=V is the number of voxels or vertices in a ROI.  The construction of the PHATE diffusion geometry is summarized as steps 1–4 here and described in more detail elsewhere^7,8^: 

1. Calculate Euclidean distance matrix between data pairs (pairs of participants), where: 

https://www.codecogs.com/eqnedit.php?latex=D(i%2Cj)%3D%7C%20%7C%20%7Bx%7D_%7Bi%7D-%7Bx%7D_%7Bj%7D%7C%20%7B%7C%20%7D%5E%7B2%7D

2. Convert D from a distance matrix into a local affinity matrix K using an adaptive bandwidth Gaussian kernel.

https://www.codecogs.com/eqnedit.php?latex=%7B%7B%7B%5Cmathcal%7BK%7D%7D%7D%7D(x%2Cy)%3D%5Cfrac%7B%7B%7B%7B%5Cmathcal%7BG%7D%7D%7D%7D(x%2Cy)%7D%7B%5Cparallel%20%7B%7B%7B%5Cmathcal%7BG%7D%7D%7D%7D(x%2C%5Ccdot%20)%7B%5Cparallel%20%7D_%7B1%7D%5E%7B%5Calpha%20%7D%5Cparallel%20%7B%7B%7B%5Cmathcal%7BG%7D%7D%7D%7D(y%2C%5Ccdot%20)%7B%5Cparallel%20%7D_%7B1%7D%5E%7B%5Calpha%20%7D%7D%2C%5Cquad%20%7B%7B%7B%5Cmathcal%7BG%7D%7D%7D%7D(x%2Cy)%3D%7B%5Cmathrm%7Be%7D%7D%5E%7B-%5Cfrac%7B%5Cparallel%20x-y%7B%5Cparallel%20%7D%5E%7B%5Calpha%20%7D%7D%7B%5Csigma%20%7D%7D

3. Row-normalize K to define transition probabilities into the *N* × *N* row stochastic matrix, *P.*

https://www.codecogs.com/eqnedit.php?latex=P(i%2Cj)%3D%5Cfrac%7B%7B%7B%7B%5Cmathcal%7BK%7D%7D%7D%7D(x_i%2C%20x_j)%7D%7B%5Cparallel%20%7B%7B%7B%5Cmathcal%7BK%7D%7D%7D%7D(x%2C%5Ccdot%20)%7B%5Cparallel%20%7D_%7B1%7D%7D

4. Use probabilities P for a Markovian random-walk diffusion process and compute the diffusion timescale *tD*, which specifies the number of steps taken in the random walk, based on the von Neumann entropy of the diffusion operator. Perform the *tD*-step random walk over *P* by raising *P* to the *tD* power. Then, compute the diffusion potential distance *PD* between all pairs of distributions at row *i* and row *j*: 

https://www.codecogs.com/eqnedit.php?latex=%7BP%7D_%7BD%7D(i%2Cj)%3D%5Csqrt%7B%5Cmathop%7B%5Csum%7D%5Climits_%7Bk%7D%7B%5Cleft(%5Clog%20(%7BP%7D%5E%7B%7Bt%7D_%7BD%7D%7D(i%2Ck)-%7BP%7D%5E%7B%7Bt%7D_%7BD%7D%7D(j%2Ck))%5Cright.%7D%5E%7B2%7D%7D

Given a matrix https://www.codecogs.com/eqnedit.php?latex=F%20%3D%20f_1%2C%20f_2%2C%20%5Cldots%20f_n where https://www.codecogs.com/eqnedit.php?latex=f_n%20%5Cin%20%7B%7B%5Cmathbb%7BR%7D%7D%7D%5E%7BN%7D for participant https://www.codecogs.com/eqnedit.php?latex=n is a https://www.codecogs.com/eqnedit.php?latex=k-dimension vector, and https://www.codecogs.com/eqnedit.php?latex=k is the number of exogenous (matched samples of a different measurement) features one opts to include in the second view. The E-PHATE procedure extends beyond the original PHATE formulation in two more steps:

5. Calculate the an affinity matrix https://www.codecogs.com/eqnedit.php?latex=A as the Euclidean distance between data pairs (pairs of participants) across feature vectors, and normalize the value to be between 0 and 1, where 1 corresponds to maximal similarity: 

https://www.codecogs.com/eqnedit.php?latex=A(i%2C%20j)%20%3D%20%5Cfrac%7B1%7D%7B1%2B%5Cleft%5C%7Cf_i%20-%20f_j%20%5Cright%5C%7C%7D

6. Convert the affinity matrix https://www.codecogs.com/eqnedit.php?latex=A into the transition probability matrix https://www.codecogs.com/eqnedit.php?latex=P_A by row-normalizing the affinity matrix A, as in step 3.

7. Combine https://www.codecogs.com/eqnedit.php?latex=P_A with the result of step 4 https://www.codecogs.com/eqnedit.php?latex=P_D via alternating diffusion: 		

https://www.codecogs.com/eqnedit.php?latex=P%3DP_AP_T

8. Embed with metric multidimensional scaling (MDS) into https://www.codecogs.com/eqnedit.php?latex=M dimensions, where https://www.codecogs.com/eqnedit.php?latex=M%3D2–3 for visualization or higher for downstream analysis.

This dual-diffusion step allows E-PHATE to learn data geometry across the neuroimaging dimensions (voxels or surface vertices) and across the dimensions of environmental metrics. Then, the dual-diffusion matrix is embedded with MDS scaling into 20 dimensions. For consistency, we compare E-PHATE embeddings with 20-D PHATE embeddings and the full voxel resolution data.

# Supplemental figures

## Figure S1: Association with CBCL subscale scores at baseline timepoint

Aggression and rule-breaking are subscales of the CBCL externalizing problems. Anxious/depressed, withdrawn/depressed, and somatic problems are subscales of the CBCL internalizing problems. Bars represent average correlation between model predicted and true problem scores on held-out participants’ data. Error bars represent the 95% confidence interval of the mean across 20 cross-validation folds. 

~ p < 0.1, * p < 0.05, ** p < 0.01, *** p < 0.001

## Figure S2: E-PHATE using 5 control features compared with the 5 social environment features

E-PHATE embeddings were computed for exogenous measures hypothesized to be unrelated to psychopathology (control measures: weight, height, age in months, number siblings, handedness) versus the five variables describing the social environment. E-PHATE embeddings were associated with psychopathology only when the exogenous variables included social environment information, not the control variables. Bars represent average correlation between model predicted and true problem scores for held-out participants’ data. Error bars represent the 95% confidence interval of the mean across 20 cross-validation folds. 

~ p < 0.1, * p < 0.05, ** p < 0.01, *** p < 0.001

## Figure S3. Comparisons of different social environment factors included in E-PHATE and associations with psychopathology. 

Bars represent average correlation between model predicted and true problem scores on held-out participants’ data. Patterns represent the factors of the social environment included in the E-PHATE calculation, where ADI =Area Deprivation Index,  FES = Family conflict   
Error bars represent the 95% confidence interval of the mean across 20 cross-validation folds. 

~ p < 0.1, * p < 0.05, ** p < 0.01, *** p < 0.001

**References**

1.	Casey, B. J. *et al.* The Adolescent Brain Cognitive Development (ABCD) study: Imaging acquisition across 21 sites. *Dev Cogn Neurosci* vol. 32 43–54 (2018).

2.	Clark, D. B. *et al.* Biomedical ethics and clinical oversight in multisite observational neuroimaging studies with children and adolescents: The ABCD experience. *Dev. Cogn. Neurosci.* **32**, 143–154 (2018).

3.	Zucker, R. A. *et al.* Assessment of culture and environment in the Adolescent Brain and Cognitive Development Study: Rationale, description of measures, and early data. *Dev. Cogn. Neurosci.* **32**, 107–120 (2018).

4.	Fan, C. C. *et al.* Adolescent Brain Cognitive Development (ABCD) study Linked External Data (LED): Protocol and practices for geocoding and assignment of environmental data. *Dev. Cogn. Neurosci.* **52**, 101030 (2021).

5.	Hagler, D. J. *et al.* Image processing and analysis methods for the Adolescent Brain Cognitive Development Study. *NeuroImage* **202**, 116091 (2019).

6.	Conley, M. I. *et al.* Executive Network Activation Moderates the Association between Neighborhood Threats and Externalizing Behavior in Youth. *Res. Child Adolesc. Psychopathol.* **51**, 789–803 (2023).

7.	Moon, K. R. *et al.* Visualizing structure and transitions in high-dimensional biological data. *Nat. Biotechnol.* **37**, 1482–1492 (2019).

8.	Busch, E. L. *et al.* Multi-view manifold learning of human brain-state trajectories. *Nat. Comput. Sci.* 1–14 (2023) doi:10.1038/s43588-023-00419-0.
