# Supplementary material for: Manifold Learning Uncovers Nonlinear Interactions between the Adolescent Brain and the Social Environment in Predicting Mental Health Problems
Source: bioRxiv. 2024 Mar 6:2024.02.29.582854. Preprint. [Version 1] doi: 10.1101/2024.02.29.582854 (PMC10942356; doi:10.1101/2024.02.29.582854)
Supplement: Supplementary file 1 [file NIHPP2024.02.29.582854V1-supplement-1.pdf]

bioRxiv preprint doi: https://doi.org/10.1101/2024.02.29.582854; this version posted March 6, 2024. The copyright holder for this preprint (which was not certified by peer review) is the author/funder, who has granted bioRxiv a license to display the preprint in perpetuity. It is made available under aCC-BY 4.0 International license.

# Supplemental figures

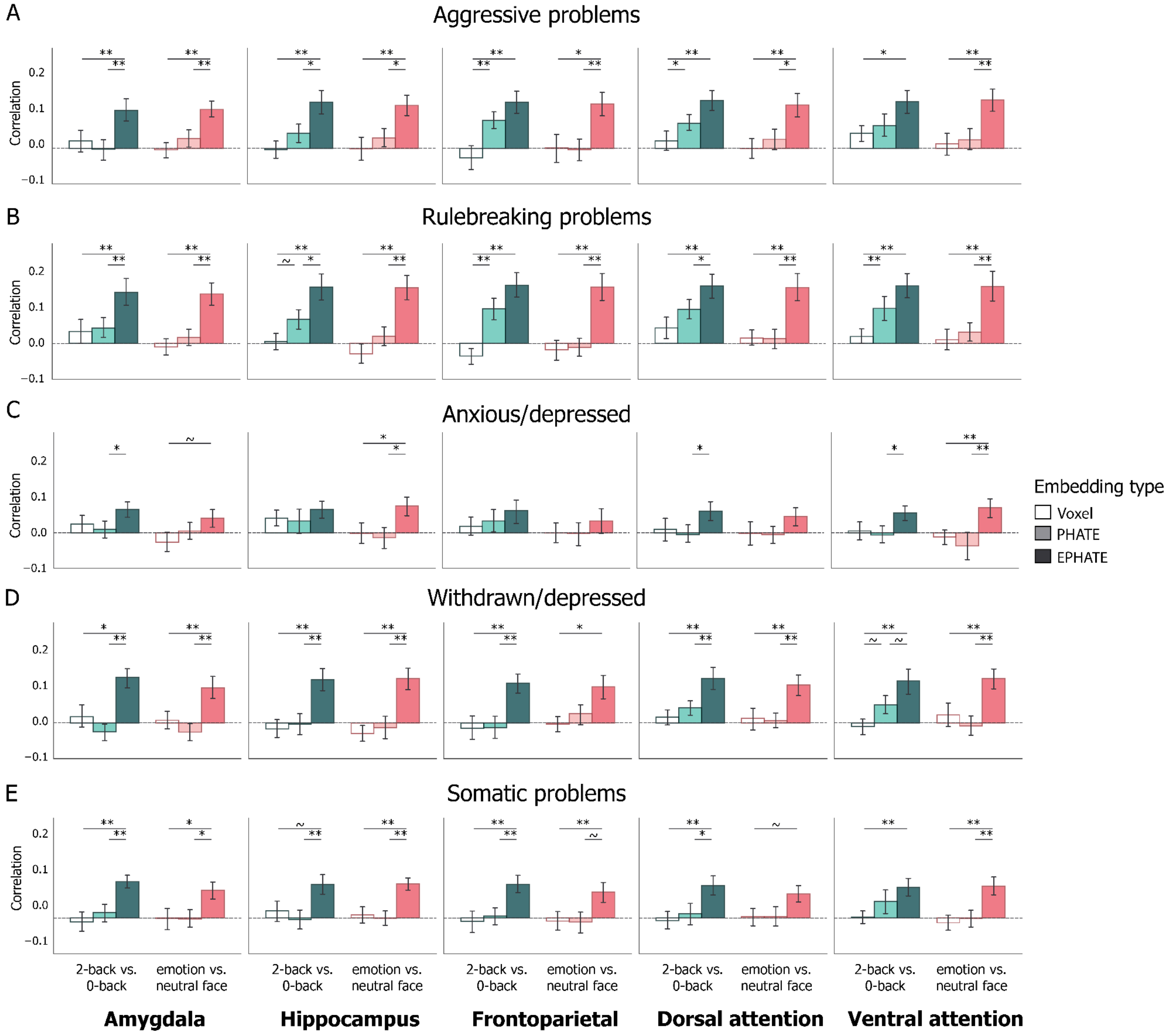


*Figure S1: Association with CBCL subscale scores at baseline timepoint*

Aggressive and rulebreaking are subscales of the CBCL externalizing problems. Anxious/depressed, withdrawn/depressed, and somatic problems are subscales of the CBCL internalizing problems. Bars represent average correlation between model predicted and true problem scores on held-out participants' data, Error bars represent the 95% confidence interval of the mean across 20 cross-validation folds.

~ $p < 0.1$, * $p < 0.05$, ** $p < 0.01$, *** $p < 0.001$

bioRxiv preprint doi: https://doi.org/10.1101/2024.02.29.582854; this version posted March 6, 2024. The copyright holder for this preprint (which was not certified by peer review) is the author/funder, who has granted bioRxiv a license to display the preprint in perpetuity. It is made available under aCC-BY 4.0 International license.

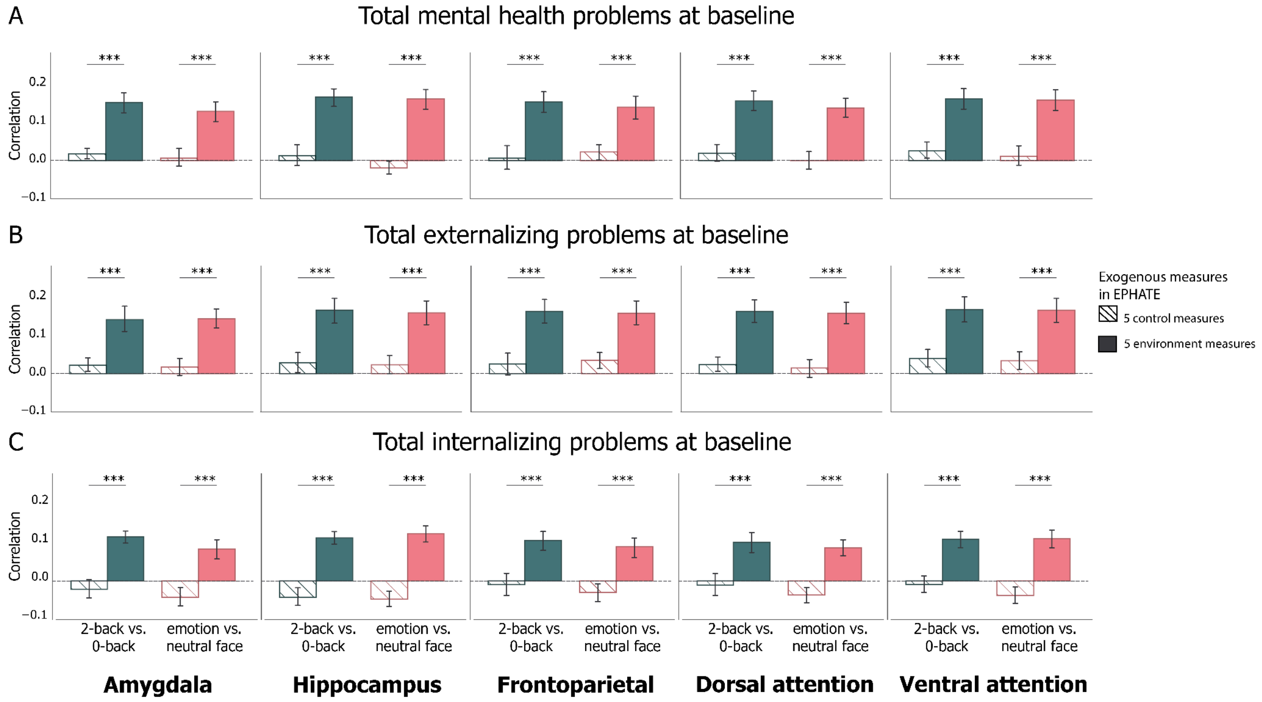


*Figure S2: EPHATE using 5 control features compared with the 5 environmental features*
EPHATE embeddings were computed for exogenous measures hypothesized to be unrelated to mental health (control measures: weight, height, age in months, # siblings, handedness) versus the five variables describing the social environment. EPHATE embeddings were associated with mental health problems only when the exogenous variables included social environment information, not the control variables. Bars represent average correlation between model predicted and true mental health problem scores for held-out participants' data. Error bars represent the 95% confidence interval of the mean across 20 cross-validation folds.
~ $p < 0.1$, * $p < 0.05$, ** $p < 0.01$, *** $p < 0.001$

bioRxiv preprint doi: https://doi.org/10.1101/2024.02.29.582854; this version posted March 6, 2024. The copyright holder for this preprint (which was not certified by peer review) is the author/funder, who has granted bioRxiv a license to display the preprint in perpetuity. It is made available under aCC-BY 4.0 International license.

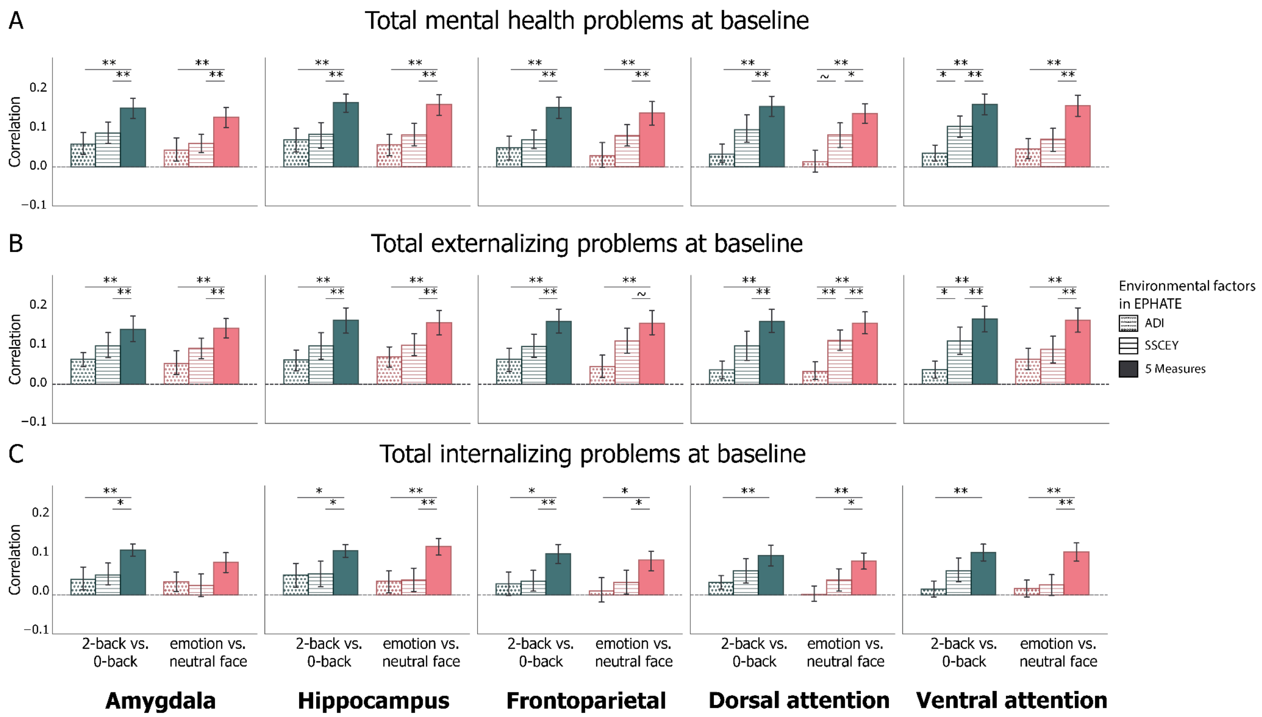


*Figure S3. Comparisons of different social environment factors included in EPHATE and associations with mental health problems.*

Bars represent average correlation between model predicted and true CBCL scores on held-out participants' data. Patterns represent the factors of the social environment included in the EPHATE calculation, where ADI =Area Deprivation Index, FES = Family conflict Error bars represent the 95% confidence interval of the mean across 20 cross-validation folds.

~ $p < 0.1$, * $p < 0.05$, ** $p < 0.01$, *** $p < 0.001$
